# Zebrafish Chromosome 14 Gene Differential Expression in the *fmr1*^*hu*2787^ Model of Fragile X Syndrome

**DOI:** 10.3389/fgene.2021.625466

**Published:** 2021-05-31

**Authors:** Karissa Barthelson, Lachlan Baer, Yang Dong, Melanie Hand, Zac Pujic, Morgan Newman, Geoffrey J. Goodhill, Robert I. Richards, Stephen M. Pederson, Michael Lardelli

**Affiliations:** ^1^School of Biological Sciences, University of Adelaide, Adelaide, SA, Australia; ^2^Bioinformatics Hub, University of Adelaide, Adelaide, SA, Australia; ^3^Queensland Brain Institute, University of Queensland, Brisbane, QLD, Australia; ^4^School of Mathematics and Physics, University of Queensland, Brisbane, QLD, Australia

**Keywords:** fragile X syndrome, zebrafish, transcriptional adaptation, transcriptome analysis, FMR1, chromosome evolution, linkage disequilibrium, homeostasis

## Abstract

Zebrafish represent a valuable model for investigating the molecular and cellular basis of Fragile X syndrome (FXS). Reduced expression of the zebrafish *FMR1* orthologous gene, *fmr1*, causes developmental and behavioural phenotypes related to FXS. Zebrafish homozygous for the hu2787 non-sense mutation allele of *fmr1* are widely used to model FXS, although FXS-relevant phenotypes seen from morpholino antisense oligonucleotide (morpholino) suppression of *fmr1* transcript translation were not observed when hu2787 was first described. The subsequent discovery of transcriptional adaptation (a form of genetic compensation), whereby mutations causing non-sense-mediated decay of transcripts can drive compensatory upregulation of homologous transcripts independent of protein feedback loops, suggested an explanation for the differences reported. We examined the whole-embryo transcriptome effects of homozygosity for *fmr1^*hu*2787^* at 2 days post fertilisation. We observed statistically significant changes in expression of a number of gene transcripts, but none from genes showing sequence homology to *fmr1*. Enrichment testing of differentially expressed genes implied effects on lysosome function and glycosphingolipid biosynthesis. The majority of the differentially expressed genes are located, like *fmr1*, on Chromosome 14. Quantitative PCR tests did not support that this was artefactual due to changes in relative chromosome abundance. Enrichment testing of the “leading edge” differentially expressed genes from Chromosome 14 revealed that their co-location on this chromosome may be associated with roles in brain development and function. The differential expression of functionally related genes due to mutation of *fmr1*, and located on the same chromosome as *fmr1*, is consistent with R.A. Fisher’s assertion that the selective advantage of co-segregation of particular combinations of alleles of genes will favour, during evolution, chromosomal rearrangements that place them in linkage disequilibrium on the same chromosome. However, we cannot exclude that the apparent differential expression of genes on Chromosome 14 genes was, (if only in part), caused by differences between the expression of alleles of genes unrelated to the effects of the *fmr1^*hu*2787^* mutation and made manifest due to the limited, but non-zero, allelic diversity between the genotypes compared.

## Background

Fragile X Syndrome (FXS) is an X-chromosome-linked form of human inherited intellectual disability affecting, on a pan-ethnic basis, approximately 1 in every 2,400 male foetuses (Owens et al., 2018). (As females are diploid for the X-chromosome, the proportion of female foetuses affected is far lower.) FXS most commonly arises due to expansion of a CGG trinucleotide repeat sequence in the human *FMR1* gene that codes for an RNA-binding protein involved in multiple aspects of RNA metabolism (Salcedo-Arellano et al., 2020). The trinucleotide repeat expansion promotes methylation and repression of *FMR1* transcription (Pieretti et al., 1991). However, other forms of mutation that reduce gene expression can cause the syndrome (Quan et al., 1995; Hammond et al., 1997). The syndrome includes non-cognitive features such as craniofacial changes, macroorchidism, and abnormalities of connective tissue, blood, and pigmentation (reviewed by Salcedo-Arellano et al., 2020). The syndrome is complicated by the existence of subtle cognitive effects due to limited expansion of the trinucleotide repeat known as a “premutation” (Salcedo-Arellano et al., 2020). The syndrome also displays the phenomenon of anticipation whereby phenotype severity increases in successive generations due to expansion of the trinucleotide repeat beyond a premutation range (Sutherland et al., 1991).

Numerous models of FXS in animals as divergent as *Drosophila* and mice have been constructed to facilitate research where access to human brain material is restricted or not possible (reviewed in Dahlhaus, 2018). As expected, all these models have their limitations. For example, mouse models of trinucleotide repeat expansion in the *Fmr1* gene show reduced FMRP protein expression but do not show increased methylation or reduced mRNA levels (Brouwer et al., 2007). For this reason, research is dominated by null mutation models and, conveniently, the mouse *Fmr1* gene is located on the mouse X-chromosome. Mouse *Fmr1* gene knockout models show effects on cognition, postsynaptic dendritic spines, craniofacial changes, and macroorchidism (The Dutch-Belgian Fragile X Consorthium, 1994; Kooy et al., 1996; Heulens et al., 2013; Dahlhaus, 2018). Mouse and zebrafish models of *FMR1* mutation have revealed impaired processing of visual (Goel et al., 2018) and auditory (Constantin et al., 2020) information, respectively, and are systems in which testing of therapeutics offers hope of ameliorating FXS (Chen et al., 2011; Dinday and Baraban, 2015; Wiley et al., 2017; Goel et al., 2018).

In zebrafish, the *FMR1* orthologous gene, *fmr1*, is autosomal so that analysis of loss-of-function phenotypes requires examination of homozygous mutants or embryos injected with morpholino antisense oligonucleotides (morphants). We published the first analysis of *fmr1* morphant zebrafish embryos in 2006 (Tucker et al., 2006). We described changes in axonal branching that could be rescued by treatment with the mGluR antagonist 2-methyl-6-(phenylethynyl)pyridine (MPEP), similar to observations of mGluR effects in mouse and *Drosophila* analyses of *FMR1* activities. We also described changes in craniofacial structure in zebrafish *fmr1* morphants. Subsequently, den Broeder and colleagues described isolation of a null mutation in *fmr1*, allele hu2787, from a TILLING (targeted induced local lesions in genomes) mutation screen (den Broeder et al., 2009). They did not observe neurite branching or craniofacial defects (or any other *FMR1*-related phenotypes) and asserted their model to be superior to analysis of *fmr1* morphants, arguing that the morphant phenotypes were likely artefactual. Analysis of the *fmr1*^*hu*2787^ mutant now dominates FXS modelling in zebrafish and, fortunately, subsequent analyses of this mutant have described numerous syndrome-relevant phenotypes (Ng et al., 2013; Shamay-Ramot et al., 2015; Marsden et al., 2018; Constantin et al., 2020).

Kok et al. (2015) summarised the puzzling, frequent discordance between phenotypes caused by gene mutations compared to morphant phenotypes caused by reduction of gene expression due to injection of morpholinos. Rossi et al. (2015), then described the phenomenon of “genetic compensation” (now referred to as “transcriptional adaptation” Kontarakis and Stainier, 2020) as contributing to this discordance. As elaborated in a subsequent paper from that laboratory (El-Brolosy et al., 2019), non-sense-medicated decay (NMD) of transcripts with premature termination codons can (in a manner independent of protein feedback loops) increase the abundance of transcripts of genes with homologous sequences that, presumably, are partially functionally redundant and ameliorate the effects of the mutation. The discovery of this phenomenon raises questions regarding the definition of “null” mutant phenotypes and reveals that reducing gene expression using morpholinos may, in some cases, provide more focussed functional effects at the molecular level that are simpler to interpret than those caused by mutations inducing NMD.

During the billions of years of biological evolution on Earth, there has been enormous selective pressure for the development of robustly stable cellular/physiological systems (homeostasis) that can survive and reproduce in the face of constant environmental and genetic change (mutation). Transcriptional adaptation is one example of a broadly acting homeostatic mechanism. Transcriptome analysis can reveal the genome-wide effects of mutations in particular genes. When a mutated gene normally functions in cellular systems controlling transcription or transcript stability, some of the consequent, genome-wide changes in transcript abundance or structure (“differential expression” of genes) may represent the direct, deleterious effects of the mutation on gene expression. However, the majority of gene expression changes detected in transcriptome analysis likely represent homeostatic responses to maintain the cellular/physiological functions in which the mutated gene normally acts.

We were curious to investigate the effects of *fmr1* mutation on gene expression and whether the homeostatic transcriptional adaptation phenomenon (detected as increased abundance of transcripts from genes containing homologous sequences) might contribute to the reported relatively milder phenotype of homozygosity for the hu2787 mutant allele of *fmr1* compared to that reported for morpholino-injected embryos. Therefore, we compared the transcriptomes of batches of entire 2 days post fertilisation (dpf) embryos homozygous for the hu2787 allele with those of batches of wild type embryos of the same age. This age was selected as a time when *fmr1* is known to be expressed (Thisse and Thisse, 2004; van ’t Padje et al., 2005) and near the end of embryogenesis (Kimmel et al., 1995) but within the developmental interval examined in the den Broeder and Tucker et al papers (Tucker et al., 2006; den Broeder et al., 2009). We observed statistically significant changes in transcript levels for *fmr1* and 20 other genes. However, significantly altered expression of transcripts from genes containing sequences with homology to the *fmr1* transcript were not observed. Curiously, 12 of the 21 genes observed to be differentially expressed, including *fmr1*, are located on Chromosome 14. Bioinformatic analysis of gene expression in the hu2787 homozygous embryos implied effects on lysosomal function and glycosphingolipid biosynthesis consistent with observations in mouse models of *Fmr1* mutation. This suggests that the colocation of genes on Chromosome 14 affected by the *fmr1*^*hu*2787^ mutation may be associated with their coaction with *fmr1* in brain development and function. The differential expression of functionally related genes located on the same chromosome as *fmr1* is consistent with R.A. Fisher’s assertion that the selective advantage of co-segregation of particular combinations of alleles of genes will favour evolutionary chromosomal rearrangements that place them in linkage disequilibrium on the same chromosome. However, due to the structure of the mating scheme used to produce the batches of homozygous mutant and wild type embryos examined, we cannot exclude that some of the apparent differential gene expression observed reflects the differential expression of different alleles of genes and is unrelated to any effects of the *fmr1*^*hu*2787^ mutation.

## Materials and Methods

### Collection of Zebrafish Embryos

The *fmr1^*hu*2787^* mutation was originally described by den Broeder et al. (2009). All the zebrafish embryos used in this work were descendants of a population of *fmr1^*hu*2787^* heterozygous and homozygous zebrafish larvae imported from the laboratory of Howard Sirotkin. Heterozygous individuals selected from this population were crossed to produce homozygous, heterozygous and non-mutant (*fmr1*^+/+^) progeny. From these progeny, pairs of homozygous and non-mutant fish were mated to produce the clutches of embryos used for transcriptome analysis. Each clutch was derived from a different pair of parents. At 2 dpf, embryos were dechorionated in E3 embryo medium and then chilled on ice for 15 min. The E3 medium was then removed and approximately 100 embryos were added to 5 volumes of RNAlater^TM^ Stabilisation Solution (Invitrogen, Carlsbad, CA, United States) at 4°C, chilled on ice for 1 h, and then stored overnight at 4°C. The tissue was then stored at −20°C until purification of RNA or DNA.

The recessive w2 allele of the gene *mifta* (*nacre*) (Lister et al., 1999) was subsequently discovered to have been present (in a heterozygous state) in some individuals of the original, imported population and was present in some of the clutches of embryos examined. None of the parents of the clutches used for transcriptome analysis were homozygous for the w2 allele. *Post hoc* analysis showed that the presence of this allele did not greatly affect the transcriptome analysis (see Supplemental Data File 2). Some of the *fmr1^*hu*2787/hu2787^* parents used to produce embryos for qPCR analysis of relative chromosome copy number were also homozygous for the w2 allele of *mitfa*, but any *mitfa^*w*2^*^/^*^*w*2^* embryos (i.e., lacking melanotic pigmentation) were removed from these clutches before analysis.

### RNA Extraction From Larval Clutches

Total RNA was isolated from four *fmr1* homozygous mutant clutches (labelled “S2,” “S4,” “S5,” “S8”) and four wild type clutches (“A,” “D,” “G,” “L”) of 2 dpf embryos using the *mir*Vana miRNA isolation kit (Thermo Fisher Scientific Inc., Waltham, MA United States). A sample size of *n* = 4 was chosen based on our experience with transcriptome analyses of zebrafish brains (Newman et al., 2019; Hin et al., 2020a,b). RNA isolation was performed according to the manufacturer’s protocol. First, the larval clutch was lysed in a denaturing lysis solution. The lysate was then extracted once with acid-phenol:chloroform leaving a semi-pure RNA sample. The sample was then purified further over a glass-fibre filter to yield total RNA. DNases were removed from the RNA samples using a DNA-free^TM^ kit (Ambion, Austin, TX, United States). To prevent degradation of samples prior to RNA sequencing, RNA was stabilised using RNAstable (Biomatrica, San Diego, CA, United States) as per the manufacturer’s protocol. Briefly, RNA was resuspended in DEPC-treated water, applied directly into an RNAstable tube and dried with a vacuum concentrator. Total RNA was then delivered to Novogene (Hong Kong, China) to assess RNA quality and for subsequent RNA sequencing.

### RNA-seq Analysis

Strand-specific, paired end (150 bp insert), polyA + library preparation and RNA sequencing (RNA-seq) was conducted by Novogene using the Illumina HiSeq 4000 platform (Illumina Inc., San Diego, CA, United States). Base-calling was performed using Illumina Casava (v1.8). We evaluated the quality of the supplied demultiplexed fastq files by fastQC (Andrews, 2010) and ngsReports (Ward et al., 2019). We then pre-processed the reads before alignment by removing adapters from any reads derived from RNA fragments less than 300 bp, removing bases from the ends of reads when the quality score fell below 20, and discarding reads less than 35 bp in length after trimming of adapters using AdapterRemoval v2.2.1 (Schubert et al., 2016). The remaining reads were then aligned to the zebrafish genome [Ensembl Release 94 (GRCz11)] using STAR v2.5.3a (Dobin et al., 2013). Aligned reads were counted using featureCounts from the Subread package (v1.5.2) (Liao et al., 2014) only if they were unique and mapped to strictly exonic regions.

### Gene Differential Expression and Gene Set Enrichment Analyses

Analysis of count data was performed using R (Zhao and Tan, 2006). We only retained genes which had more than 1 count in 4 or more of the 8 RNA-seq libraries, leaving 18,280 genes for downstream analysis. We performed differential gene expression analysis using the generalised linear model capabilities and likelihood-ratio tests from the package edgeR (Robinson et al., 2010), after calculation of normalisation offsets using Conditional Quantile Normalisation (Hansen et al., 2012). We considered genes to be differentially expressed (DE) due to homozygosity for the hu2787 allele of *fmr1* if the false discovery rate (FDR)-adjusted *p*-value was less than 0.05. Complete results of this differential expression analysis are shown in Supplementary Data File 1. We tested for over-representation of pre-defined gene sets within the DE genes using goseq (Young et al., 2010), using a weighted average GC content as input for the probability weighting function (PWF). The gene sets we used were the KEGG (v7.1) and HALLMARK (v7.1) gene sets available from MSigDB (Liberzon et al., 2011). We downloaded the gene sets as a .gmt file with human Entrez gene identifiers and converted these to zebrafish Ensembl IDs using a mapping file downloaded from BioMart (Smedley et al., 2015). We also tested whether there was over-representation of genes on any of the 26 chromosomes (i.e. including the mitochondrial chromosome) in the DE gene lists with goseq. We considered a gene set to be over-represented in the DE genes if the Bonferroni-adjusted *p*-value was < 0.05.

To obtain a more complete view on the changes to gene expression due to *fmr1* genotype, we performed the self-contained, fast rotation gene set testing method fry (Wu et al., 2010) on the cqn-adjusted logCPM values.

### Implementation of the *RNAseq Short Variant Discovery (SNPs* + *Indels)* Workflow

Variant calls were generated for each sample at sites across the genome where there was sufficient evidence for a non-reference nucleotide in at least one sample. Variants were determined based on the Genome Analysis Toolkit (GATK) best practices workflow (Van der Auwera and O’Connor, 2020) for *RNAseq short variant discovery (SNPs + Indels)*. Briefly, trimmed reads were aligned to the GRCz11 (Ensembl release 94) genome using STAR v2.7.7a (Dobin et al., 2013) two-pass mode to achieve better alignments around novel splice junctions. PCR duplicates were marked using Picard (Broad Institute, (n.d.), see References) such that only independent observations were counted, and reads that spanned introns were split into multiple supplementary alignments with the GATK v4.2 (Van der Auwera and O’Connor, 2020) SplitNCigar tool to allow for downstream processing. Before base quality score recalibration, a set of known variants was generated by the bootstrapping method described in GATK’s best practices. This involved an initial round of variant calling with the GATK v4.2 HaplotypeCaller tool on the unrecalibrated alignments. Variants with a minimum phred-scaled confidence threshold of 20 were assigned as the set of known variants for base recalibration, which was achieved with the GATK v4.2 BaseRecalibrator tool. A final round of HaplotypeCaller was run on the recalibrated alignments and the resulting variant calls were filtered based on GATK’s recommended specific hard filters (phred-scaled *p*-value using Fisher’s exact test for strand bias (FS) > 30, variant confidence/quality by depth (QD) > 2). Lastly, variants were selected for only single nucleotide polymorphisms using the GATK v4.2 SelectVariants tool. See also Supplementary Data File 4.

### Analyses to Detect Transcriptional Adaptation

We obtained the list of genes with sequences similar to *fmr1* by performing a BLASTn search using the Ensembl BLAST server with the *fmr1* cDNA sequence as input (i.e., the mRNA sequence including untranslated regions). Firstly, we queried this sequence against the zebrafish (GRCz11, Ensembl version 101) reference transcriptome with parameters set for distant homology. Detected homologous sequences with an *E*-value < 1 were tested to determine whether they were upregulated due to *fmr1* genotype. To determine if *fmr1* shares homology with other features shown to influence transcriptional adaptation, such as promoters and introns (El-Brolosy et al., 2019), the *fmr1* cDNA sequence was similarly queried against the entire zebrafish genome (GRCz11, Ensembl version 101). This returned no additional information. An exploration of similarity thresholds was performed similar to that of El-Brolosy et al. (2019) (see Methods section “Sequence similarity and subsampling analyses”). *p*-values were computed by bootstrapping random subsamples. Thresholds were chosen such that each threshold included at least one additional unique alignment. No significant correlations between sequence similarity and upregulation of expression were found at any threshold for any of the three parameters analysed (alignment length, bit score, and *E*-value, see Supplementary Data File 5).

### DNA Extraction for Relative Standard Curve Quantitative PCR

Three clutches of 2 dpf zebrafish embryos homozygous for *fmr1*^*hu*2787^ were collected. Each clutch contained approximately 100 embryos. Three batches of around 100 wild type zebrafish embryos were generated from three different tanks of wild type zebrafish families and collected at 2 dpf. Genomic DNA (gDNA) was extracted and purified using the Wizard^®^ genomic DNA purification kit (Promega, Madison, Wisconsin, United States). Genomic DNA (gDNA) concentrations were estimated using a NanoDrop Microvolume Spectrophotometer (Thermo Fisher Scientific). Each 25 μL qPCR reaction contained 40 ng of gDNA, 0.2 μM of each PCR primer and Power SYBR green master mix PCR solution (Applied Biosystems, Thermo Fisher Scientific). The relative standard curve method was used for quantification to determine the amounts of *fmr1*, *psen1* and *rpl13* in wild type samples relative to *fmr1*^*hu*2787^ homozygous samples. The standard curve was generated by a serial dilution, having 40, 20, and 10 ng of wild type gDNA per reaction. The qPCR was performed on an ABI 7000 Sequence Detection System (Applied Biosystems) using a 96-well plate. The amplification consisted of a holding stage and a cycling stage. The holding stage was 50°C for 2 min and then 95°C for 10 min, and the cycling stage had 40 cycles of 95°C for 15 s and 60°C for 1 min. Three technical PCR replicates were performed on each DNA sample and their mean was used to represent the quantity. The quantities of the genes *fmr1* and *psen1* were calculated relative to *rpl13*. Raw quantitative PCR data are shown in Supplementary Data File 1. The PCR primers used and the relative chromosome ratios calculated are described in Supplementary Data File 3.

## Results and Discussion

### Differential Gene Expression in *fmr1*^*hu*2787/*hu*2787^ vs. + / + Embryos at 2 dpf

Transcriptome (RNA-seq) analysis was conducted on clutches of wild type and *fmr1*^*hu*2787^ homozygous mutant embryos (*n* = 4 clutches for each genotype) that had been allowed to develop for 2 dpf at ∼28.5°C. Each clutch of embryos was the product of a unique pair mating (i.e., no clutches shared any parents).

**FIGURE 1:**
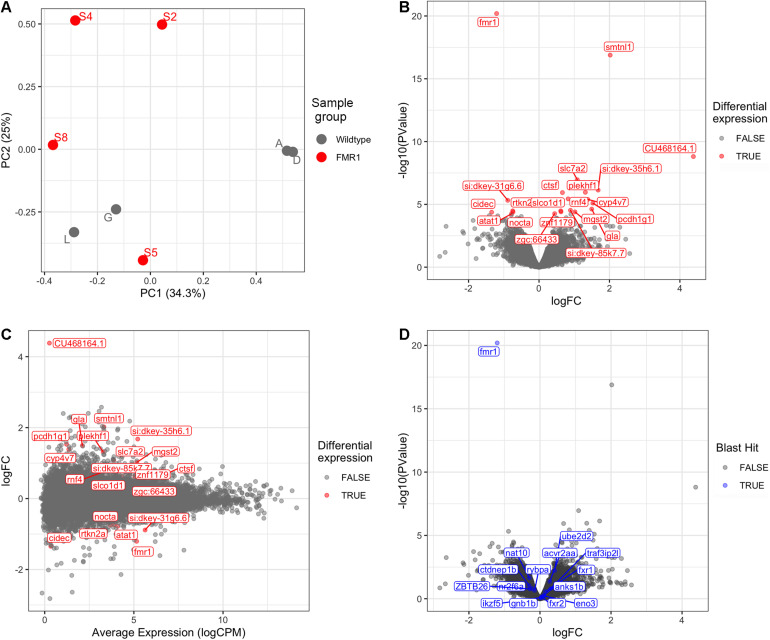
Transcriptome analysis of *fmr1*^*hu*2787/*hu*2787^ vs. wild type entire embryos at 2 dpf. **(A)** Principal Component (PC) analysis of larval wild type (A, D, G, L) and *fmr1*^*hu*2787/*hu*2787^ (S2, S4, S5, S8) RNA-seq data. **(B)** Volcano plot of differential gene expression. **(C)** Mean-difference (MD) plot showing the average levels of expression of RNAs plotted against fold change differences in expression between mutant and wild type embryos. **(D)** Volcano plot of differential expression as in b, but indicating genes sharing some degree of sequence homology with *fmr1* (indicated in blue). No significantly increased expression is seen.

Bioinformatic analysis is described in detail in Supplementary Data File 2 and the results are summarised here. Principal Component Analysis (Figure 1A) did not suggest clearly discrete clustering of genotypes which was reflected in the paucity of differentially expressed genes detected (Figures 1B,C and see below).

### Genes Differentially Expressed at 2 dpf Due to Homozygosity for *fmr1*^*hu*2787^

Using a False Discovery Rate (FDR) of 0.05, only 21 genes were detected as differentially expressed (DE, see Table 1). Levels of *fmr1* transcripts in the mutant embryos were approximately half of that in the wild type embryos consistent with non-sense-mediated decay (NMD) due to the premature termination codon generated by the *fmr1*^*hu*2787^ mutation. While NMD has not been demonstrated formally for transcripts of this allele (by stabilisation through inhibition of translation) and neither do we do so here, the location of the stop codon more than 80 nucleotides upstream of the next exon/exon boundary is consistent with NMD activity (Nagy and Maquat, 1998).

**TABLE 1:**
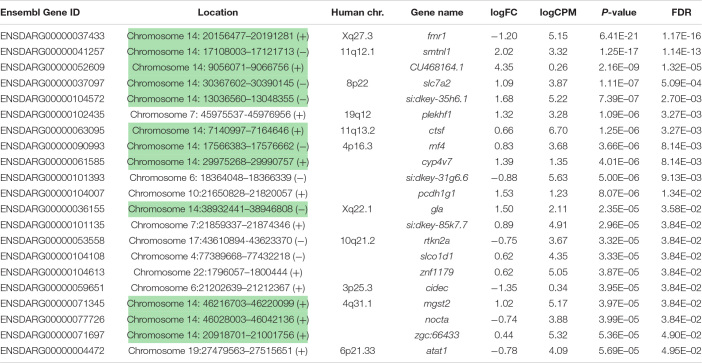
Genes differentially expressed in 2 dpf homozygous *fmr1*^*hu*2787^ embryos.

**Gene positions on zebrafish chromosomes are taken from reference genome assembly GRCz11. Those on Chromosome 14 are highlighted in green. “(+)” denotes the nominal forward strand and “(*−*)” the reverse strand of chromosomes. Where human orthologous genes are known, their chromosomal locations from GRCh38 are given under “Human chr.” Log_2_ values are given for Fold Change (FC) and Counts Per Million (CPM).**

**FIGURE 2:**
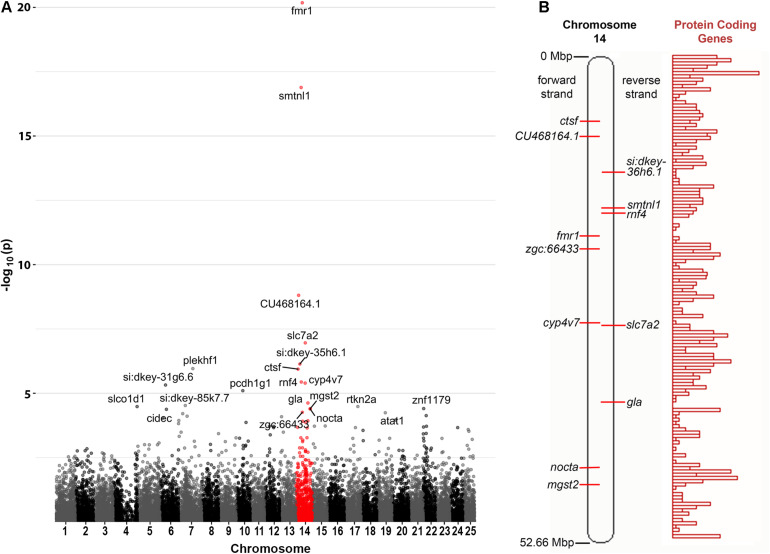
Chromosome distribution of DE genes. **(A)** Manhattan plot of *p*-values for differential expression of genes on the chromosomes of zebrafish. An enrichment for DE genes on Chromosome 14 is apparent with possible enrichment also for genes on Chromosome 22 (see also Supplementary Data File 2). **(B)** A diagram of zebrafish Chromosome 14 taken from https://asia.ensembl.org (Yates et al., 2020) and including a representation of relative protein coding gene density along the chromosome. Loci for the Chromosome 14 genes found to be DE in *fmr1*^*hu*2787/*hu*2787^ 2 dpf embryos are indicated (see also Table 1).

Curiously, we observed that 12 of the 21 genes found to be DE at a false discovery rate (FDR) of less than 0.05 are located on zebrafish Chromosome 14. This was found to be a highly significant over-representation by goseq (Bonferroni adjusted *p*-value < 2 × 10^–16^, see Figure 2A and Supplementary Data File 2). This included genes both up- and down-regulated (Table 1) at loci widely dispersed on the chromosome and transcribed from either DNA strand (Figure 2B). As Chromosome 14 (DRE13 in Phillips et al., 2006) has a p arm/entire chromosome length ratio of 0.27 (Phillips et al., 2006) it is likely that these loci are distributed on both chromosome arms.

One possible explanation for many genes on one chromosome appearing differentially expressed would be if the *fmr1*^*hu*2787^ mutation was associated with meiotic or mitotic instability of Chromosome 14, although this would not be consistent with the simultaneous up- and down-regulation of Chromosome 14 genes observed. Nevertheless, to test this possibility we conducted quantitative PCR on genomic DNA extracted from an additional three groups each of 2 dpf *fmr1*^*hu*2787/*hu*2787^ and +/+ embryos. We used PCR primers amplifying genomic DNA sequences from the genes *fmr1* (Chromosome 14), *psen1* (Chromosome 17) and *rpl13* (Chromosome 7). No difference was observed in the amplification of *fmr1* sequence compared to *rpl13* and the amplification of *psen1* sequence relative to *rpl13* in clutches of either *fmr1*^*hu*2787/*hu*2787^ or + / + embryos (Table 2) supporting that the stability of Chromosome 14 is normal (see also Supplementary Data File 3).

**TABLE 2:** PCR primer pairs used to quantify relative genomic DNA copy number.

Gene	Chromosomal location (ENSEMBL)	PCR primer sequences	Mean ratio gene/*rpl13*	
			+/+	*fmr1*/*fmr1*
***fmr1***	Chromosome 14: 20,156,477–20,191,281 forward strand.	5′-TCCAGGACCAGGAGGCTGTA-3′	0.99	0.98
		5′-CCCACTCTTGCCAATGACTTTTC-3′		
***psen1***	Chromosome 17: 51,206,225–51,224,800 reverse strand.	5′-CTACACACAGAAGGACGGACAGC-3′	0.98	0.93
		5′-CCATCCCTAAACTGCTCCTACT-3′		
***rpl13***	Chromosome 7: 67,467,702–67,477,495 forward strand.	5′-TGGAATGGATGAATAGGTTTTTA-3′	1	1
		5′-CTCTCTTCTGCCAGTCTTTATGA-3′		

### Is the Enrichment for DE Genes on Chromosome 14 an Allelic Expression Artefact?

Enrichments for DE genes on the same chromosome as a mutation have been observed previously. For example, in a study of neural crest development in zebrafish, Dooley et al. (2019) saw this for embryos homozygous for mutations in *sox10* (Chromosome 3) or *mitfa* (Chromosome 6). However, they were unable to distinguish whether this was due to reduced genetic variation around the homozygous loci (allowing genes with alleles having divergent levels of expression to be observed as differentially expressed) or due to real regulatory effects of the mutations. To find evidence possibly supporting one or the other of these alternatives in our analysis of *fmr1*^*hu*2787/*hu*2787^ embryos compared to wild type embryos, we examined allelic diversity within the homozygous mutant and wild type transcriptome data sets. We implemented the “*RNAseq short variant discovery (SNPs* + *Indels)*” workflow to identify and quantify exonic single nucleotide polymorphism (SNP) variants in the DE genes on Chromosome 14 and other chromosomes. As expected, this did identify the absence of allelic diversity at Chromosome 14 DE genes proximal to *fmr1* in the *fmr1*^*hu*2787/*hu*2787^ embryos while some of the Chromosome 14 DE genes more distal to *fmr1* showed greater allelic diversity (see Supplementary Data File 4). Greater, although still limited, allelic diversity was seen for all the Chromosome 14 DE gene transcripts in the wild type embryos and for most of the non-Chromosome 14 DE gene transcripts in both *fmr1*^*hu*2787/*hu*2787^ and wild type embryos. Overall, the generally moderate levels of allelic diversity observed for the DE genes of our analysis do not allow us to exclude that many of the genes identified as DE on Chromosome 14 are displaying differences in allelic expression levels rather than responses to mutation of *fmr1*.

Interestingly, transcripts of the non-Chromosome 14 DE genes *cidec* and *plekhf1* showed variation between genotypes but no variation within genotypes supporting the possibility that their apparent differential expression may reflect allelic expression differences rather than responses to mutation of *fmr1* (see Supplementary Data File 4).

A function-based explanation for why mutation of *fmr1* might cause enriched differential expression of Chromosome 14 genes may lie in selective pressure during evolution for homeostatic robustness combined with low rates of meiotic recombination along zebrafish chromosomes. Indeed, meiotic recombination is suppressed in male zebrafish so that most chromosomes have recombination distance lengths of less than 50 centiMorgans (Singer et al., 2002). This means that, in zebrafish male meiosis, no two genes on Chromosome 14 will show completely independent assortment of alleles. If a fitness advantage existed for co-segregation of particular pairs of alleles of functionally related genes, then chromosomal rearrangements leading to the co-location of those alleles within 50 centiMorgans on the same chromosome might be under positive selection during evolution. This phenomenon was first suggested by RA Fisher in 1930 in his book, *The genetical theory of natural selection* (Fisher, 1930). In discussing pairs of alleles (“factors”) of functionally interacting genes in linkage disequilibrium he stated, “…*the presence of pairs of factors in the same chromosome, the selective advantage of each of which reverses that of the other, will always tend to diminish recombination, and therefore to increase the intensity of linkage in the chromosomes of that species.*”

Similar selective forces may have driven the accumulation of genes important for neural function during evolution of the human X-chromosome (for which meiotic recombination is limited in human males) (Skuse, 2005; Nguyen and Disteche, 2006) and within which the human *FMR1* gene resides. This idea is consistent with the observation by Salih and Adelson (2009) that particular Gene Ontology (GO) terms, when mapped to genes spanning the bovine genome, appear clustered. The clustering of GOs was subsequently observed in the genomes of other species (Tiirikka et al., 2014). Also, numerous examples exist of genome rearrangements during evolution leading to clustering of functionally related genes (e.g., Qi et al., 2004; Wong and Wolfe, 2005; Hill et al., 2019). There is also evidence that chromosomal rearrangements (inversions) experience natural selection to maintain associations between functionally related alleles (Said et al., 2018).

If limited (but non-zero) allelic diversity in transcriptome analyses of genotypes such as we have performed here can generate significant levels of apparent differential gene expression unrelated to the genotypes of interest, then that is an important constraint on interpretation of transcriptome data. It is important that future work examine the extent to which limited allelic diversity can cause the appearance of such gene differential expression between genotypes.

### No Evidence for Increased Expression of Gene Transcripts With Homology to *fmr1*

The phenomenon of transcriptional adaptation may contribute to explaining the frequent disparity between embryo development phenotypes caused by loss of gene function due to inhibition of transcript splicing/translation by morpholino antisense oligonucleotides and loss of gene function due to gene mutation (Kok et al., 2015). El-Brolosy et al. (2019) showed that a transcript subject to NMD could cause increased abundance of transcripts from genes possessing sequences with homology to that transcript. To test whether transcriptional adaptation might contribute to the reported differences in developmental phenotypes observed between *fmr1*^*hu*2787/*hu*2787^ embryos (den Broeder et al., 2009) and embryos injected with morpholinos inhibiting *fmr1* transcript translation (Tucker et al., 2006), we searched the zebrafish genome and transcriptome (GRCz11, Ensembl version 101) for features with sequence homology to *fmr1* cDNA. These are listed in Supplementary Data File 5. None of these genes were significantly differentially expressed in our comparison of *fmr1*^*hu*2787/*hu*2787^ and +/+ larval clutches (Figure 1D).

Testing for the enrichment of groups of genes (gene sets) in transcriptome data sets can provide more sensitive detection of significant changes in expression than examining genes individually. Therefore, we defined as a set the genes identified with homology to *fmr1* and subjected this set to the gene set testing method fry (Wu et al., 2010) (from the limma package Ritchie et al., 2015) with a directional hypothesis (increased transcript abundance). This gave a *p*-value for enrichment of 0.6 which does not support that the expression of this set of genes is increased.

Our analyses have not been able to detect transcriptional adaptation due to homozygosity for the *fmr1*^*hu*2787^ allele. However, we cannot exclude that NMD of *fmr1*^*hu*2787^ allele transcripts might induce this phenomenon. It is possible that the restricted expression of *fmr1* in 2 dpf embryos limits the sensitivity of our analyses to detect differential expression of genes with homology to *fmr1* by diluting their relative abundance with transcripts from cells not expressing *fmr1*. Alternatively, the transcriptional adaptation phenomenon may not function for the *fmr1* gene at this particular developmental stage, although this seems unlikely considering the supposed generality of the phenomenon.

Interestingly, unlike for the *fmr1*^*hu*2787^ allele, a recently published loss-of-function mutation in *fmr1* induced using the CRISPR-Cas9 system and that deletes the translation start codon does produce craniofacial abnormalities (Hu et al., 2020). However, these abnormalities appear more subtle than reported by Tucker et al. (2006) for morpholino inhibition of *fmr1* translation. Also, in work submitted for publication, careful re-examination of craniofacial morphology in larvae homozygous for the *fmr1*^*hu*2787^ mutation itself does, in fact, reveal structural differences compared to wild type larvae (Zhu et al., 2021). Therefore, the differences between the phenotypic effects of the *fmr1*^*hu*2787^ mutation and injection of morpholinos blocking *fmr1* mRNA translation may not be as extensive as originally believed.

### Prediction of Cellular Functions Affected by Homozygosity for *fmr1*^*hu*2787^

To gain insight into the cellular functions that might be affected by the *fmr1*^*hu*2787^ mutation we performed a functional analysis within the set of DE genes using goseq (Young et al., 2010) with the HALLMARK and KEGG gene sets as defined in MSigDBv7.2 via the R package msigdbr (Dolgalev, 2020). The requisite probability weighting function (PWF), for the probability of a gene being considered DE, was estimated using the average GC content across all transcripts of a gene. We found that the KEGG gene sets for *lysosome* and *glycosphingolipid biosynthesis globo series* were significantly over-represented in the DE genes, although only one or two DE genes were driving this over-representation. The apparent effect of zebrafish *fmr1* mutation on lysosomal function is consistent with observations that loss of *Fmr1* gene function in mice affects lysosome function (Yan et al., 2018).

We also performed the gene set testing method fry using chromosome position, HALLMARK and KEGG gene sets to obtain a more complete view of the changes to gene expression due to *fmr1* genotype. No significant gene sets were observed. However, the genes on Chromosome 14 were those closest to being significantly altered as a group with an FDR adjusted *p*-value of 0.16.

We investigated further the possible functional significance of changes to expression of genes from Chromosome 14 by performing an over-representation analysis of those genes driving the enrichment of the set of Chromosome 14 genes (the “leading edge” genes from gene set enrichment analysis, GSEA, see Supplementary Data File 2). Relative to all the detected genes in the RNA-seq analysis, the leading edge Chromosome 14 genes were significantly over-represented in the KEGG gene sets *neuroactive ligand receptor interactions* and *RNA polymerase*. Interestingly, the *neuroactive ligand receptor interactions* gene set was previously observed to be enriched in the cerebellum of 8–10 week old male *Fmr1* knockout mice compared to wild type mice (Kong et al., 2014) and this is consistent with FXS as a developmental neuropathology. The enrichment of genes from this gene set among the leading edge genes expressed from zebrafish Chromosome 14 suggests that their co-location on this chromosome may reflect selective pressure during evolution for linkage disequilibrium between advantageous alleles of functionally related genes.

### Limitations

Our ability to identify a number of differentially expressed genes, some at very small FDR corrected *p*-values despite moderate fold changes in expression, supports our confidence that we would have been able to detect transcriptional adaptation (increased abundance of transcripts from genes possessing homologous sequences) if it was induced by the hu2787 allele of *fmr1* at 2 dpf. However, the small number of gene sets identified as statistically significantly enriched in *fmr1^*hu*2787^* homozygous mutants may reflect the limited sample size used. It is also conceivable that transcriptional adaptation effects on transcript abundance are dependent on developmental stage and that we did not examine the transcriptome at an appropriate time to observe these effects. Future studies using larger sample sizes and examining a range of developmental stages would give improved definition of enriched gene sets (suggesting altered cellular functions) and would also likely identify additional genes on Chromosome 14 with expression affected by mutation of *fmr1*.

## Conclusion

Despite the apparent NMD of *fmr1*^*hu*2787^ transcripts, and the reported milder developmental phenotype of *fmr1*^*hu*2787^ homozygotes relative to individuals in which the function of this gene is suppressed using morpholinos, we did not see evidence for transcriptional adaptation by increased transcription of genes possessing sequences with homology to *fmr1*, at least at 2 dpf. This illustrates possible variability of the occurrence of the recently discovered transcriptional adaptation mechanism, and that more research is required to understand the factors modulating it. Our analysis has revealed the accumulation on zebrafish Chromosome 14 of genes for which expression is affected by mutation of *fmr1*. This may be the result of selection over evolutionary time for robust cellular homeostasis together with linkage between particular combinations of alleles of functionally related genes. Loss of *fmr1* gene function in the zebrafish *fmr1*^*hu*2787^ model produces similar effects on the brain transcriptome to loss of the orthologous gene in mice, and supports the translatability to humans of discoveries regarding *fmr1* function made using zebrafish.

## Data Availability Statement

All data described in this paper are available within the paper itself, or in the Supplementary Data Files. The RNA-seq data are available at the Gene Expression Omnibus (GEO) database under Accession Number GSE151443. The code used to perform this analysis is available at https://github.com/UofABioinformaticsHub/20190129_Lardelli_FMR1_RNASeq and at https://github.com/baerlachlan/210305_fmr1_snv.

## Ethics Statement

The animal study was reviewed and approved by the Animal Ethics Committee of the University of Queensland. Permit Number QBI/061/17.

## Author Contributions

ML and RIR conceived the study. GJG administered, and ZP performed, zebrafish breeding for embryo collection and preservation. MN prepared RNA for, and administered, RNA-seq analysis. YD designed, performed and analysed qPCR. SMP supervised all bioinformatics analyses. MH and LB identified genes with sequences possessing *fmr1* homology and performed initial DE gene analysis. LB performed further tests to detect transcriptional adaptation and the analysis of allelic diversity in differentially expressed genes. KB performed all other bioinformatics analysis. All authors participated in drafting the manuscript.

## Conflict of Interest

The authors declare that the research was conducted in the absence of any commercial or financial relationships that could be construed as a potential conflict of interest.

## Abbreviations

CPM: counts per million
DE: differentially expressed
FC: fold change
FDR: false discovery rate
*FMR1*: *FMRP translational regulator 1* (human gene)
*Fmr1*: mouse orthologue of human *FMR1*
*fmr1*: zebrafish orthologue of human *FMR1;*
FXS: Fragile X Syndrome
gDNA: genomic DNA
GATK: Genome Analysis Toolkit
GEO: Gene Expression Omnibus
KEGG: Kyoto Encyclopaedia of Genes and Genomes
MD: mean-difference
mGluR: metabotropic glutamate receptor
mitfa/nacre: *melanocyte inducing transcription factor a* gene
morpholino: morpholino antisense oligonucleotide
MPEP: 2-methyl-6-(phenylethynyl)pyridine
NMD: non-sense-mediated decay
PC: principal component
psen1: *presenilin 1* gene
*rpl13*: *Ribosomal Protein L13* gene
SNP: single nucleotide polymorphism
TILLING: Targeting Induced Local Lesions in Genomes.
