# Supplementary material for: Zebrafish Chromosome 14 Gene Differential Expression in the fmr1hu2787 Model of Fragile X Syndrome
Source: Front Genet. 2021 May 31;12:625466. doi: 10.3389/fgene.2021.625466 (PMC8203322; doi:10.3389/fgene.2021.625466)
Supplement: Supplementary file 2 [file Data_Sheet_2.pdf]

Supplementary Data File 2: Bioinformatics analyses

The code which generated the results in this Supplementary Data File is available at:

https://github.com/UofABioinformaticsHub/20190129_Lardelli_FMR1_RNASeq

Introduction:

Previously, a differential gene expression analysis was performed using limma (Ritchie et al., 2015;Law et al., 2016). However, in our experience, the generalised linear model (GLM) capabilities of edgeR (Robinson et al., 2010;Law et al., 2016) detect more differentially expressed genes.

Prior to count-level analysis, the initial dataset was pre-processed using the following steps:

- Adapters were removed from any reads derived from RNA fragments < 300bp
- Bases were removed from the end of reads when the quality score dipped below 20
- Reads < 35bp after trimming were discarded

After trimming alignment was performed using STAR v2.5.3a to the *Danio rerio* genome included in Ensembl Release 94 (GRCz11). Aligned reads were counted using featureCounts (Liao et al., 2014) for each gene if alignments were unique and overlapped strictly within exonic regions. Undetectable genes (genes which contained less than one counted alignment in at least 4 of the 8 samples) were excluded from the analysis.

Initial differential gene expression analysis:

We first performed an initial differential gene expression analysis using the generalised linear model functionality of edgeR. EdgeR uses a negative binomial variance function and estimates dispersions using the Cox-Reid profile-adjusted likelihood (CR) method (Robinson et al., 2010). We specified a design matrix with the wild type genotype as the intercept (or baseline) and the effect of homozygosity for the hu2787 allele of *fmr1* as a coefficient (**Table S1**).

Table S1: Design matrix used in differential expression analysis

	(Intercept)	*fmr1*$^{hu2787/hu2787}$
S2	1	1
S4	1	1
S5	1	1
S8	1	1
A	1	0
D	1	0
G	1	0
L	1	0

After dispersions were estimated and the negative binomial model was fitted, likelihood ratio tests were performed to determine which genes were significantly differentially expressed (FDR-adjusted p-value < 0.05) in the *fmr1*$^{hu2787/hu2787}$ samples. We identified 14 differentially expressed genes in this

differential expression test and these genes mostly had low/average expression levels (**Figure S2**). This bias may impact gene set enrichment analysis and should be corrected for, as gene sets with low-medium expressed genes will appear as enriched for being upregulated.

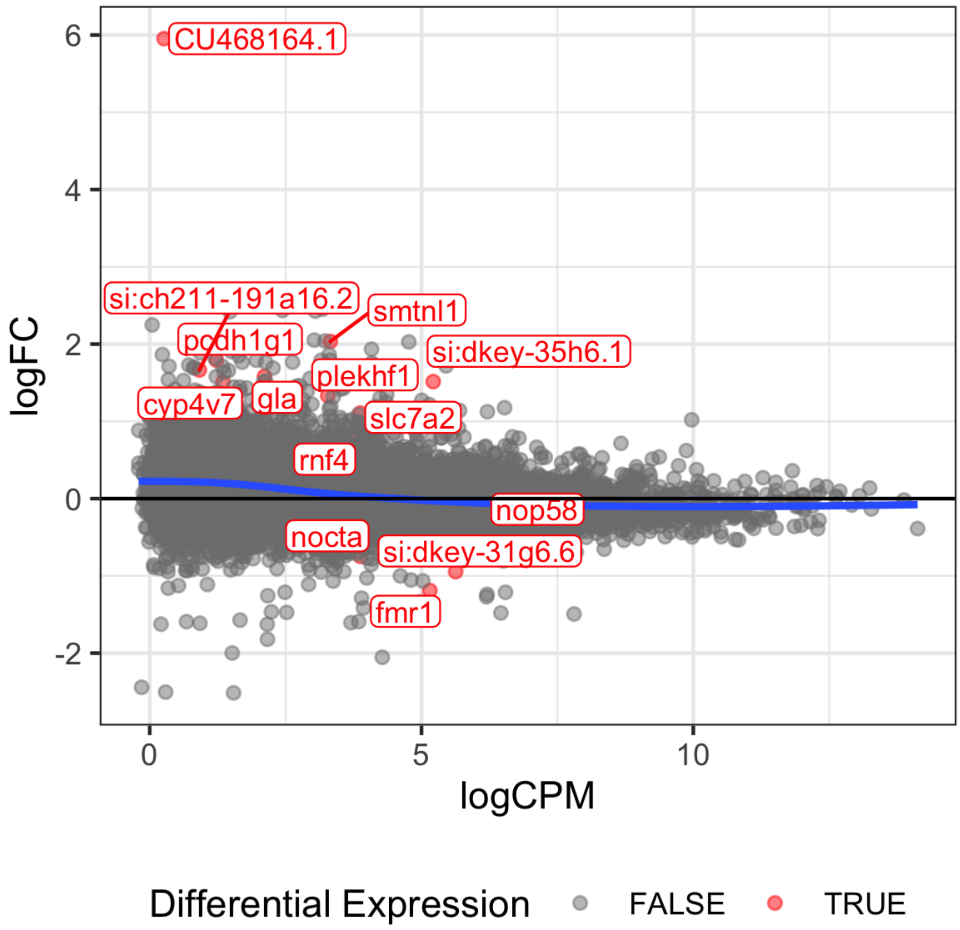

Figure S2: Mean-difference (MD) plot displaying the average expression level (logCPM) against the log_2 fold change (logFC) of each gene. Differentially expressed genes are coloured in red. The blue fitted line from a generalised additive model (gam) identified a small bias for lowly expressed genes to be upregulated.

In response to the observed bias, cqn (Hansen et al., 2012) was used to rectify this issue as it may be derived from either a length or GC artefact. GC and length information for each gene was obtained from the Ensembl database (GRCz11, release 98). For input to cqn, GC content and length were taken as weighted averages and simple averages respectively.

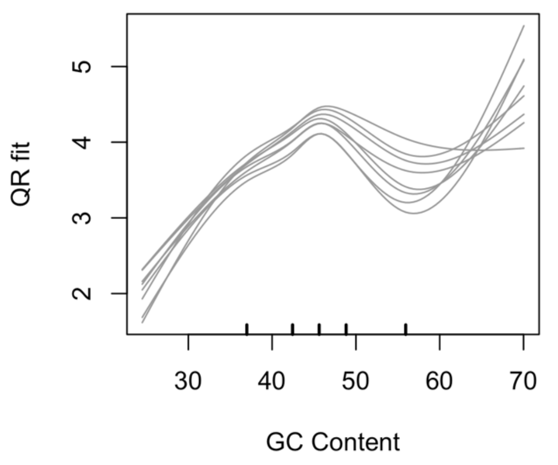

Figure S3: Model fits for GC content and gene length under the cqn model. Variability between samples is clearly visible.

We then performed an additional differential gene expression analysis, including the offset term generated by cqn when fitting the negative binomial model. The likelihood ratio tests from this model identified 21 differentially expressed genes. Comparison of the MD plots before and after cqn show that the observed bias was mostly removed, suggesting the gene GC content and length were contributing factors.

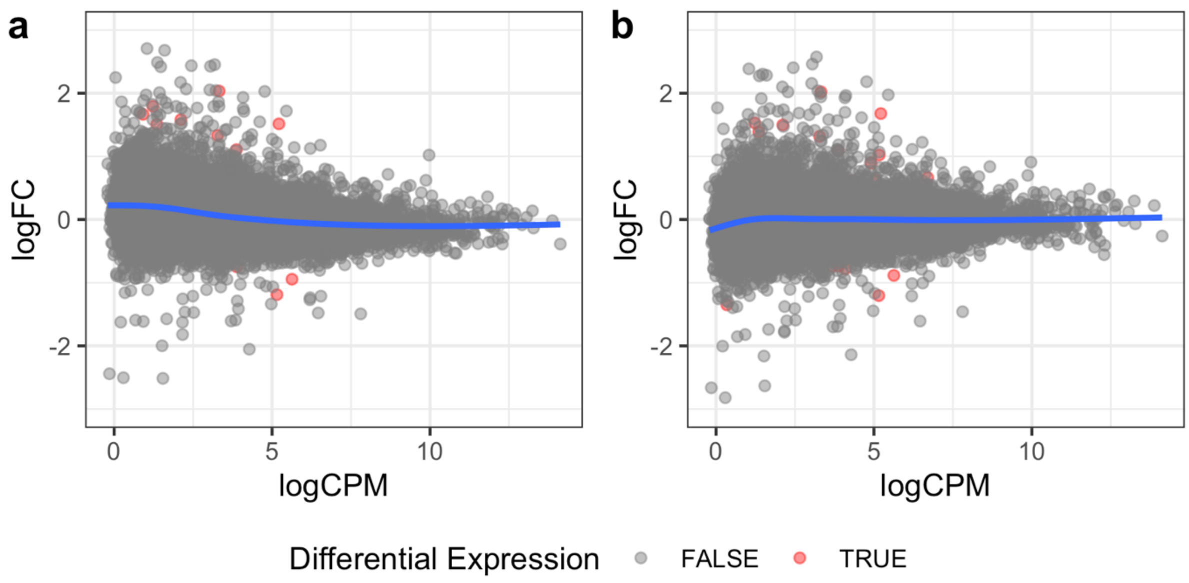

Figure S4: Comparison of mean difference plots before and after conditional quantile normalisation (cqn). The bias evident in a) the pre-cqn plots is no longer present in b) the post-cqn plot, suggesting GC and length bias were contributing factors. Blue fit lines are derived from generalised additive models.

We next tested for over-representation of genes based on which chromosome they are located on using goseq (Young et al., 2010). Goseq tests whether there is over-representation of pre-defined gene sets amongst the set of DE genes. It does not take into account the magnitude or direction of

the fold change. However, it can take into account a bias of a gene being classified DE due to its GC content. We found that genes on chromosome 14 were highly over-represented in the DE genes (**Table S2**).

**Table S2: Enrichment testing within the differentially expressed genes based on chromosomal location**					
**Chromosome**	**Expected**	**Observed**	**Gene Set Size**	**p-value**	$p_{bonferroni}$
14	0.76	12	664	~0	~0
6	0.94	2	820	0.26	1
7	1.08	2	942	0.33	1
10	0.75	1	653	0.51	1
17	0.81	1	703	0.55	1
22	0.72	1	629	0.56	1
19	0.83	1	721	0.57	1
4	0.80	1	695	0.57	1

We next tested for over-representation of the KEGG and HALLMARK gene sets within the DE gene list. We downloaded the KEGG and HALLMARK gene sets from the Molecular Signatures database (MSigDB) as a .gmt file with human gene Entrez identifiers. The human gene Entrez identifiers were converted to zebrafish Ensembl identifiers using a mapping file downloaded from the Ensembl Biomart web server (https://m.ensembl.org/biomart). Some genes in the KEGG gene sets did not contain a zebrafish orthologue. Therefore, the gene sets occasionally contained only a small number of genes and this would not be particularly informative. For this reason, only KEGG gene sets which contained > 10 zebrafish genes were retained for analysis.

We identified that the KEGG gene sets for *lysosome* and *glycosphingolipid biosynthesis globo series* were significantly over-represented in the set of DE genes. The HALLMARK gene set for *early estrogen response* approached significance for over-representation. The results from enrichment testing for HALLMARK gene sets within the set of DE genes is found in **Table S3** and the results for the KEGG gene sets are found in **Table S4**.

**Table S3: Enrichment testing for HALLMARK gene sets within the set of differentially expressed genes**					
**HALLMARK Gene Set**	**Expected**	**Observed**	**Gene Set Size**	**p-value**	$p_{Bonferroni}$
ESTROGEN RESPONSE EARLY	0.19	2	168	0.01	0.08
PROTEIN SECRETION	0.11	1	94	0.10	0.72
INFLAMMATORY RESPONSE	0.13	1	116	0.13	0.90
HALLMARK COMPLEMENT	0.18	1	155	0.16	1.00
ESTROGEN RESPONSE LATE	0.19	1	162	0.18	1.00
P53 PATHWAY	0.21	1	183	0.19	1.00
MTORC1 SIGNALING	0.23	1	198	0.21	1.00

Table S4: Enrichment testing for KEGG gene sets within the set of differentially expressed genes					
**KEGG gene set**	**Expected**	**Observed**	**GS Size**	**p-value**	$p_{bonferroni}$
LYSOSOME	0.13	2	114	0.00	0.02
GLYCOSPHINGOLIPID BIOSYNTHESIS GLOBO SERIES	0.01	1	8	0.01	0.05
GALACTOSE METABOLISM	0.02	1	20	0.01	0.11
SPHINGOLIPID METABOLISM	0.04	1	34	0.03	0.21
GLYCEROLIPID METABOLISM	0.04	1	37	0.03	0.23
DRUG METABOLISM CYTOCHROME P450	0.04	1	32	0.03	0.24
METABOLISM OF XENOBIOTICS BY CYTOCHROME P450	0.04	1	33	0.03	0.24
GLUTATHIONE METABOLISM	0.05	1	41	0.04	0.32

Next, we performed gene set enrichment analysis (GSEA) on all detectable genes in the RNA-seq experiment to obtain a more complete view on the changes to gene expression due to *fmr1* genotype. We performed GSEA using the fry (Wu et al., 2010;Ritchie et al., 2015) algorithm from the limma package. Fry approximates the ROAST method, which uses residual space rotation rather than permutations to determine the significance of a gene set showing changes to gene expression (Wu et al., 2010). Using this method, we did not find any significantly altered gene sets (KEGG, HALLMARK or chromosome position) after FDR adjustment of the mixed p-value. The top 10 results are shown in the **Table S5**.

**Table S5: Top 10 most highly ranked gene sets across chromosome position, HALLMARK and KEGG testing using fry.** Tests were non-directional (Mixed).			
**Gene set**	**Number of genes**	**p-value**	p_{FDR}
chromosome 14	664	6.1E-04	0.16
KEGG HISTIDINE METABOLISM	23	0.005	0.35
HALLMARK MYC TARGETS V2	57	0.006	0.35
KEGG VALINE LEUCINE AND ISOLEUCINE DEGRADATION	44	0.006	0.35
KEGG RNA POLYMERASE	26	0.007	0.35
KEGG GLYCOSPHINGOLIPID BIOSYNTHESIS GLOBO SERIES	8	0.008	0.35
KEGG GALACTOSE METABOLISM	20	0.009	0.35
KEGG GLYCEROLIPID METABOLISM	37	0.02	0.39
KEGG RNA DEGRADATION	54	0.02	0.39
KEGG GLYCOSYLPHOSPHATIDYLINOSITOL GPI ANCHOR BIOSYNTHESIS	22	0.02	0.39

Analysis of genes on chromosome 14:

The set of genes located on chromosome 14 was found to be over-represented in the DE list by goseq, and was the gene set identified by fry to contain the most DE genes (**Table S5**), although it did not attain the threshold for significance ($p<0.05$). We hypothesised that the most differentially expressed genes from chromosome 14 might be enriched in a particular biological pathway which could explain the over-representation detected by goseq (see Table S2 above).

To investigate this, we performed the fast implementation of the GSEA (Subramanian et al., 2005) algorithm, fgsea (Korotkevich et al., 2021), on the gene sets for chromosome position to obtain a list of the "leading edge" genes for chromosome 14. The first step for fgsea is to generate a ranked list of all genes in the experiment. We ranked genes based on the statistical significance of their differential expression (without direction). A visual representation of the ranked list is shown in **Figure S5**.

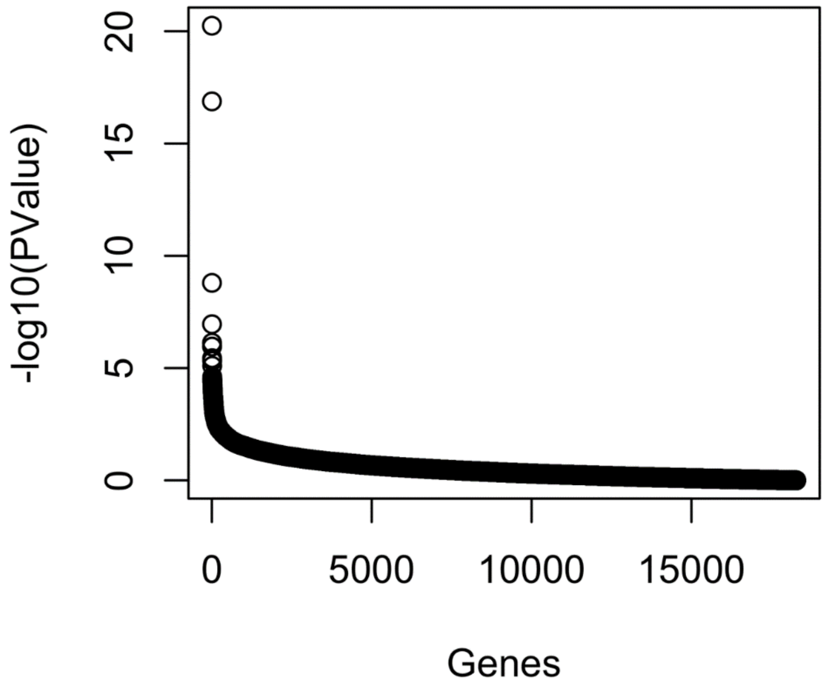

Figure S5: Visual representation of the ranked list.

All detectable genes were ranked on the statistical significance of their differential expression, resulting in the most differentially expressed genes at the start of the ranked list (i.e. smallest p-value), and the least differentially expressed genes at the end of the ranked list (largest p-value).

We used this ranked list as input for fgsea, which tests whether there is an enrichment of pre-defined gene sets at either end of the ranked list. We considered a gene set to be enriched if the Bonferroni adjusted p-value was < 0.05. **Table S6** gives the significantly enriched chromosome position gene sets identified by fgsea.

Table S6: Chromosomal position gene sets found to be significantly enriched by fgsea.					
Gene set	p-value	Bonferroni adjusted p-value	Enrichment score	Normalised enrichment score	Gene set size
Chromosome 14	1e-5	0.00026	0.53	1.398	664
Chromosome 22	2e-5	0.00052	0.46	1.2	629

The leading edge genes for each gene set from the fgsea algorithm are the most highly ranked genes of a gene set in the ranked list and contribute most to its enrichment. **Figure S6** shows the enrichment plots of genes found on chromosomes 14 and 22 relative to the ranked list. The leading edge genes are the genes which are positioned in the ranked list before the peak of the enrichment score.

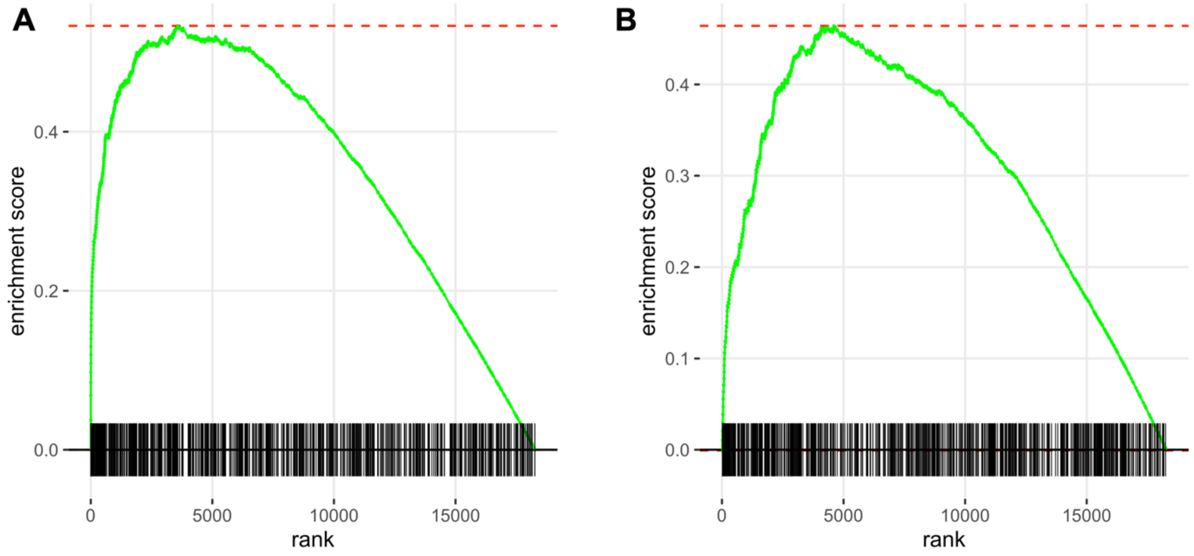

Figure S6: Enrichment plot of genes from chromosome 14 and 22.

The x-axis shows the ranked list of genes, with black bars indicating a gene from **A**) chromosome 14 and **B**) chromosome 22, and missing bars indicating a gene not from either chromosome. The y-axis gives the enrichment score, with the green line indicating the running enrichment score along the ranked list. The genes located before the peak are the leading edge subset.

To determine whether the leading edge subset of genes from chromosome 14 are enriched in any biological pathways, we performed over-representation analysis on the chromosome 14 leading edge against all detectable genes in the RNA-seq experiment. We found that the KEGG gene sets for *RNA polymerase* and *neuroactive ligand receptor interactions* were found to be significantly enriched after correction for multiple testing by FDR, but not by the more stringent Bonferroni method (**Table S7**).

Table S7: Top 7 KEGG gene sets found to be over-represented in the chromosome 14 leading edge relative to all genes detected in the RNA-seq experiment.

Gene set	p-value	p_{FDR}	p_{bonf}	No. genes in leading edge	No. genes in gene set
RNA POLYMERASE	0.00303	0.043	0.061	3	26
NEUROACTIVE LIGAND RECEPTOR INTERACTION	0.0043	0.043	0.086	6	135
AMYOTROPHIC LATERAL SCLEROSIS ALS	0.0188	0.0942	0.375	3	50
GLYCOSAMINOGLYCAN BIOSYNTHESIS HEPARAN SULFATE	0.0274	0.0942	0.547	2	23
LONG TERM POTENTIATION	0.038	0.0942	0.769	3	66
LYSOSOME	0.0401	0.0942	0.802	4	114
APOPTOSIS	0.041	0.0942	0.829	3	68

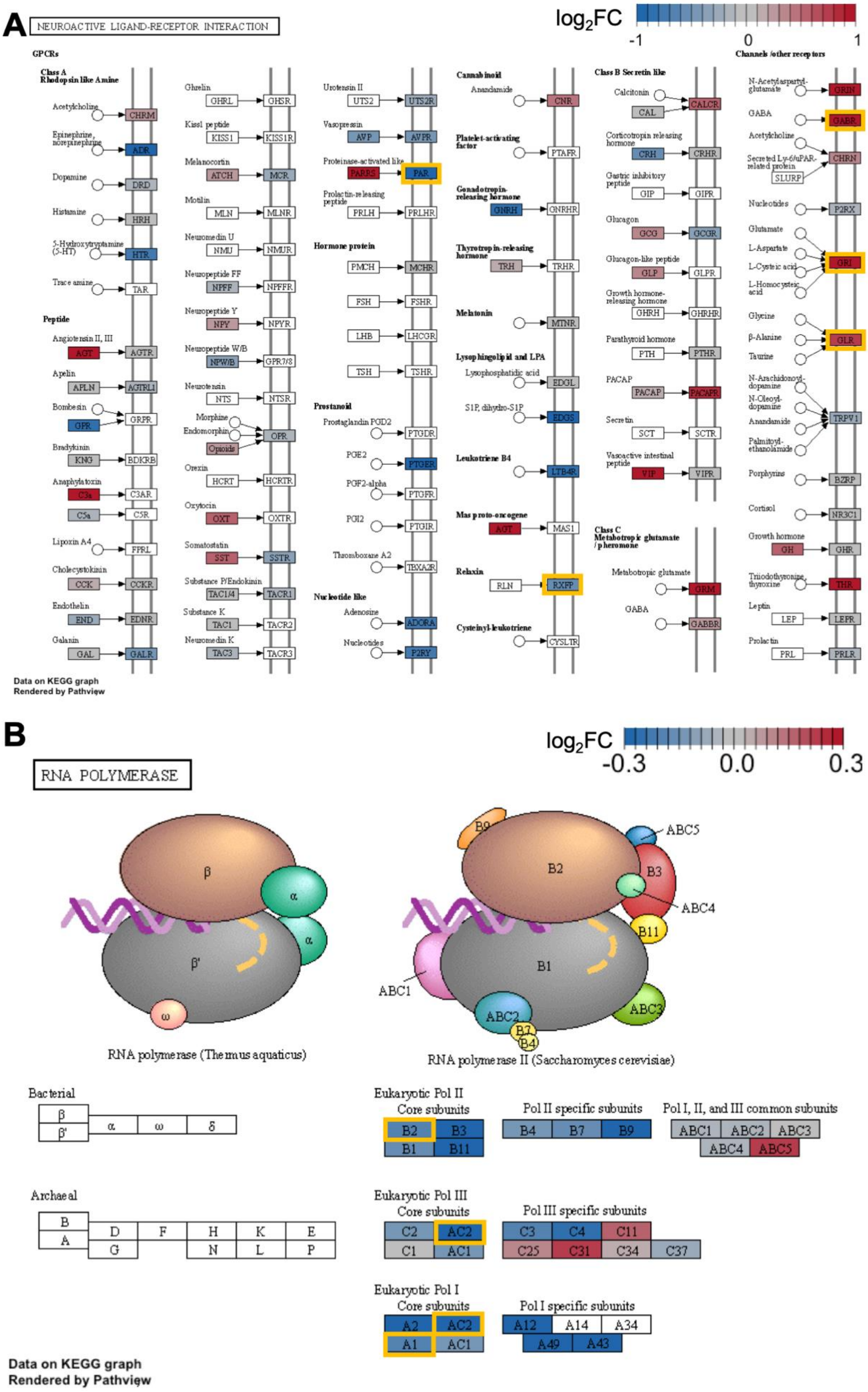

A
NEUROACTIVE LIGAND-RECEPTOR INTERACTION
log2FC
-1
0
1
GPCRs
Class A Rhodopsin like Amine
Acetylcholine
CHRM
Epinephrine, norepinephrine
ADR
Dopamine
DRD
Histamine
HRH
5-Hydroxytryptamine (5-HT)
HTR
Trace amine
TAR
Peptide
Angiotensin II, III
AGT
AGTR
Apelin
APLN
AGTRL1
Bombesin
GPR
GRPR
Bradykinin
KNG
BDKRB
Anaphylatoxin
C3a
C3AR
C5a
C5R
Lipoxin A4
FPRL
Cholecystokinin
CCK
CCKR
Endothelin
END
EDNR
Galanin
GAL
GALR
Ghrelin
GHRL
GHSR
Kiss1 peptide
KISS1
KISS1R
Melanocortin
ATCH
MCR
Motilin
MLN
MLNR
Neuromedin U
NMU
NMUR
Neuropeptide FF
NPFF
NPFFR
Neuropeptide Y
NPY
NPYR
Neuropeptide W/B
NPW/B
GPR7/8
Neurotensin
NTS
NTSR
Morphine
Endomorphin
Opioids
OPR
Orexin
HCRT
HCRTR
Oxytocin
OXT
OXTR
Somatostatin
SST
SSTR
Substance P/Endokinin
TAC1/4
TACR1
Substance K
TAC1
TACR2
Neuromedin K
TAC3
TACR3
Urotensin II
UTS2
UTS2R
Vasopressin
AVP
AVPR
Proteinase-activated like
PARRS
PAR
Prolactin-releasing peptide
PRLH
PRLHR
Hormone protein
PMCH
MCHR
FSH
FSHR
LHB
LHCGR
TSH
TSHR
Prostanoid
Prostaglandin PGD2
PTGDR
PGE2
PTGER
PGF2-alpha
PTGFR
PGI2
PTGIR
Thromboxane A2
TBXA2R
Nucleotide like
Adenosine
ADORA
Nucleotides
P2RY
Cannabinoid
Anandamide
CNR
Platelet-activating factor
PTAFR
Gonadotropin-releasing hormone
GNRH
GNRHR
Thyrotropin-releasing hormone
TRH
TRHR
Melatonin
MTNR
Lysophingolipid and LPA
Lysophosphatidic acid
EDGL
S1P, dihydro-S1P
EDGS
Leukotriene B4
LTB4R
Mas proto-oncogene
AGT
MAS1
Relaxin
RLN
RXFP
Cysteinyl-leukotriene
CYSLTR
Class B Secretin like
Calcitonin
CAL
CALCR
Corticotropin releasing hormone
CRH
CRHR
Gastric inhibitory peptide
GIP
GIPR
Glucagon
GCG
GCGR
Glucagon-like peptide
GLP
GLPR
Growth hormone-releasing hormone
GHRH
GHRHR
Parathyroid hormone
PTH
PTHR
PACAP
PACAP
PACAPR
Secretin
SCT
SCTR
Vasoactive intestinal peptide
VIP
VIPR
Class C Metabotropic glutamate / pheromone
Metabotropic glutamate
GRM
GABA
GABBR
Channels /other receptors
N-Acetylaspartyl-glutamate
GRIN
GABA
GABR
Acetylcholine
Secreted Ly-6/uPAR-related protein
SLURP
CHRN
Nucleotides
P2RX
Glutamate
L-Aspartate
L-Cysteic acid
L-Homocysteic acid
GRI
Glycine
β-Alanine
Taurine
GLR
N-Arachidonoyl-dopamine
N-Oleoyl-dopamine
Anandamide
Palmitoyl-ethanolamide
TRPV1
Porphyrins
BZRP
Cortisol
NR3C1
Growth hormone
GH
GHR
Triiodothyronine, thyroxine
THR
Leptin
LEP
LEPR
Prolactin
PRL
PRLR
Data on KEGG graph
Rendered by Pathview
B
RNA POLYMERASE
log2FC
-0.3
0.0
0.3
β
α
α
β'
ω
RNA polymerase (Thermus aquaticus)
B9
ABC5
B2
B3
ABC4
B11
B1
ABC1
ABC2
ABC3
B7
B4
RNA polymerase II (Saccharomyces cerevisiae)
Bacterial
β
β'
α
ω
δ
Archaeal
B
A
D
F
H
K
E
G
N
L
P
Eukaryotic Pol II
Core subunits
B2
B3
B1
B11
Pol II specific subunits
B4
B7
B9
Pol I, II, and III common subunits
ABC1
ABC2
ABC3
ABC4
ABC5
Eukaryotic Pol III
Core subunits
C2
AC2
C1
AC1
Pol III specific subunits
C3
C4
C11
C25
C31
C34
C37
Eukaryotic Pol I
Core subunits
A2
AC2
A1
AC1
Pol I specific subunits
A12
A14
A34
A49
A43
Data on KEGG graph
Rendered by Pathview

Figure S7: Pathview visualisation of changes to gene expression *in neuroactive ligand receptor interactions* and the *RNA polymerase* complex.

The Kyoto Encyclopedia of Genes and Genomes, KEGG, pathways for **A**, *neuroactive ligand receptor interactions* and **B**, *RNA polymerase*, with the intensity of the colour representing the $\log_2$FC of each gene. Genes which are present in the leading edge of chromosome 14 are indicated with the orange boxes. Plots are adapted from Pathview (Luo and Brouwer, 2013) and displayed with permission of KEGG, (Kanehisa and Goto, 2000).

As an alternative viewpoint, we performed over-representation analysis on the chromosome 14 leading edge subset against all genes on chromosome 14. No significant over-representation was found (**Table S8**). However, all three genes from the *ALS* and *long term potentiation* gene sets which are found on chromosome 14 are present in the leading edge subset, suggesting a possible co-regulatory mechanism.

Table S8: Top 7 KEGG gene sets found to be over-represented in the chromosome 14 leading edge relative to all genes on chromosome 14.

Gene set	p-value	p_{FDR}	p_{bonf}	No. genes in leading edge	No. genes in gene set on chr 14.
AMYOTROPHIC LATERAL SCLEROSIS ALS	0.0296	0.16	0.591	3	3
LONG TERM POTENTIATION	0.0296	0.16	0.591	3	3
ALZHEIMERS DISEASE	0.0343	0.16	0.696	4	5
FOCAL ADHESION	0.0274	0.16	1	4	6
NEUROACTIVE LIGAND RECPTOR INTERACTION	0.038	0.16	1	6	11
APOPTOSIS	0.0401	0.16	1	3	4
RNA POLYMERASE	0.041	0.16	1	3	4

Testing the influence of *w2* (*nacre*) mutation contamination

Some of the parents used to generate the clutches of larvae in this experiment were heterozygous for the *w2* allele of *melanocyte inducing transcription factor a* (*mitfa*), a C to T point mutation which results in a premature stop codon in exon 3 of *mifta* (Lister et al., 1999). (Apparently this allele was present in the original stock of *fmr1*^{hu2787} fish we imported into Australia.)

We first assessed the proportion of reads which aligned to the *w2* allele of *mifta* relative to the wild type allele to estimate the proportion of parents who carried the *w2* allele. Sample “D” and “S5” had relatively high proportions of *w2*-aligned reads. Samples S2, S4 and S8 had relatively low proportions of *w2*-aligned reads. Samples A, G and L had no reads aligned to the *w2* allele. Exploratory analysis between the proportion of reads aligning to the *w2* allele and the first four principal components of the dataset (which capture approximately 85% of the total variability) did not identify any strong correlations.

To determine whether the proportion of *w2* reads affects the results of a differential gene expression test, we used the proportion of *w2* reads as a blocking variable (as a categorical factor, with no, low and high *w2* contamination as the levels) in a limma voom analysis with the *duplicateCorrelation* function. A comparison between a ranking statistic (sign(logFC) x -log10(p-

value)) showed marginal changes to the results of the DE analysis. Therefore, the presence of the *w2* allele in *mifta* likely has minimal effects on DE analysis and can be ignored (**Figure S8**).

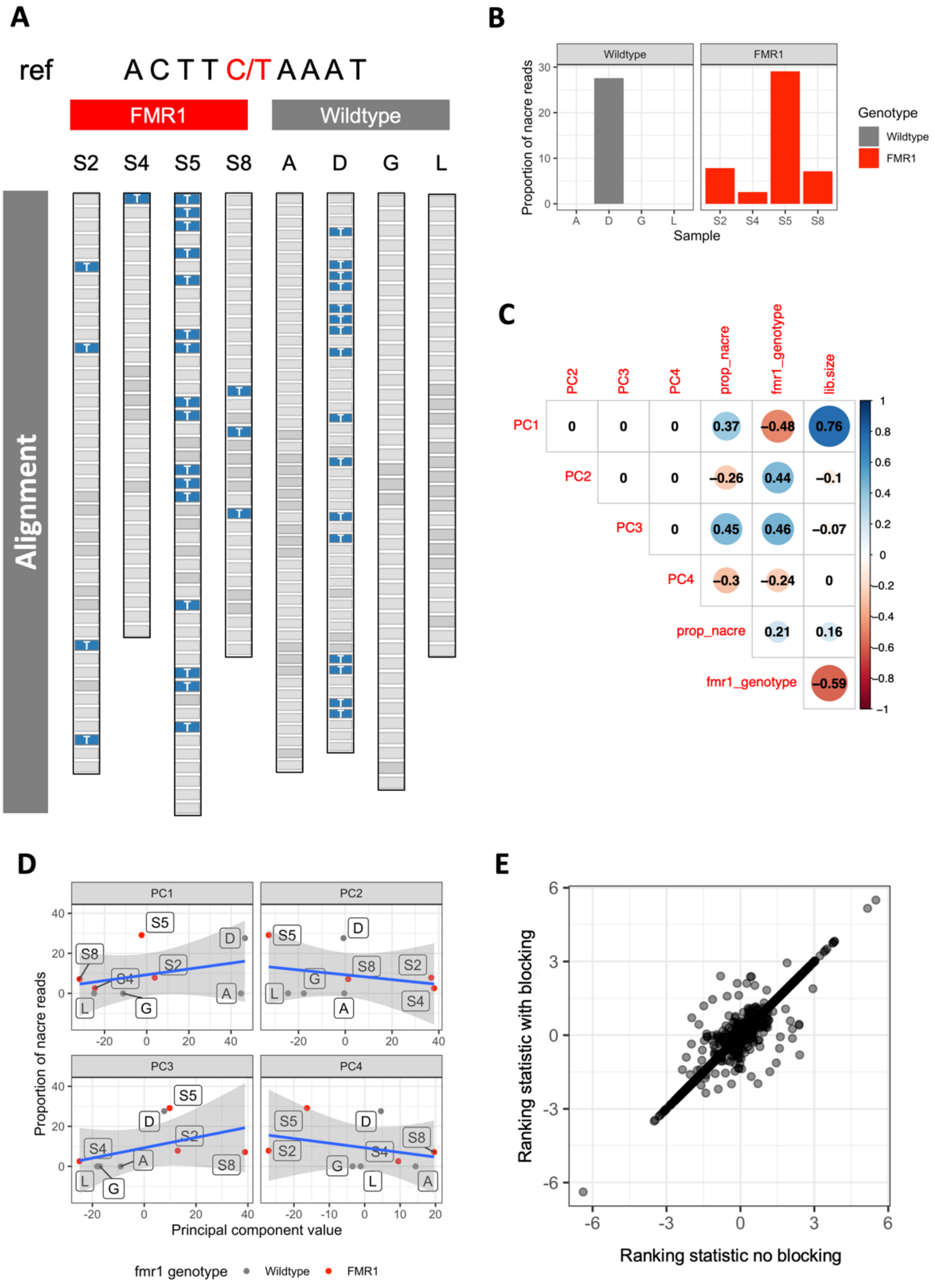

Figure S8: Presence of the *w2* allele of *mifta* does not greatly affect the transcriptomes of pooled *fmr1*$^{-/-}$ larvae.

A) Alignment of reads to the *w2* site of melanocyte inducing transcription factor a (*mitfa,* on chromosome 6, position 43429185 according to the GRCz11 build of the zebrafish genome). **B)** Percentage of reads aligning to the *w2* site for each sample. **C)** Pearson correlations between the first four principal components of the conditional-quantile normalised expression values for each gene, and the proportion of reads aligning to the *w2* site (prop_nacre), *fmr1* genotype, and RNA-seq

library size (lib.size). **D)** Proportion of *w2* (nacre) reads shown against the first four principal component values. Minimal correlation is observed. However, the standard errors (grey) for the regression lines in blue are large, suggesting that the effect is not highly significant. **E)** Scatterplot showing a ranking statistic (sign(logFC) * -log10(p-value)) for each gene with and without using the proportion of *w2* reads as a blocking variable in a limma voom analysis. The most highly ranked genes (i.e. the most differentially expressed) do not change when including the proportion of *w2* reads in the model.

References

Hansen, K.D., Irizarry, R.A., and Wu, Z. (2012). Removing technical variability in RNA-seq data using conditional quantile normalization. *Biostatistics* 13**,** 204-216.

Kanehisa, M., and Goto, S. (2000). KEGG: kyoto encyclopedia of genes and genomes. *Nucleic Acids Res* 28**,** 27-30.

Korotkevich, G., Sukhov, V., Budin, N., Shpak, B., Artyomov, M.N., and Sergushichev, A. (2021). Fast gene set enrichment analysis. *bioRxiv***,** 060012.

Law, C.W., Alhamdoosh, M., Su, S., Dong, X., Tian, L., Smyth, G.K., and Ritchie, M.E. (2016). RNA-seq analysis is easy as 1-2-3 with limma, Glimma and edgeR. *F1000Res* 5.

Liao, Y., Smyth, G.K., and Shi, W. (2014). featureCounts: an efficient general purpose program for assigning sequence reads to genomic features. *Bioinformatics* 30**,** 923-930.

Lister, J.A., Robertson, C.P., Lepage, T., Johnson, S.L., and Raible, D.W. (1999). nacre encodes a zebrafish microphthalmia-related protein that regulates neural-crest-derived pigment cell fate. *Development* 126**,** 3757-3767.

Luo, W., and Brouwer, C. (2013). Pathview: an R/Bioconductor package for pathway-based data integration and visualization. *Bioinformatics* 29**,** 1830-1831.

Ritchie, M.E., Phipson, B., Wu, D., Hu, Y., Law, C.W., Shi, W., and Smyth, G.K. (2015). limma powers differential expression analyses for RNA-sequencing and microarray studies. *Nucleic Acids Res* 43**,** e47.

Robinson, M.D., Mccarthy, D.J., and Smyth, G.K. (2010). edgeR: a Bioconductor package for differential expression analysis of digital gene expression data. *Bioinformatics* 26**,** 139-140.

Subramanian, A., Tamayo, P., Mootha, V.K., Mukherjee, S., Ebert, B.L., Gillette, M.A., Paulovich, A., Pomeroy, S.L., Golub, T.R., Lander, E.S., and Mesirov, J.P. (2005). Gene set enrichment analysis: a knowledge-based approach for interpreting genome-wide expression profiles. *Proc Natl Acad Sci U S A* 102**,** 15545-15550.

Wu, D., Lim, E., Vaillant, F., Asselin-Labat, M.L., Visvader, J.E., and Smyth, G.K. (2010). ROAST: rotation gene set tests for complex microarray experiments. *Bioinformatics* 26**,** 2176-2182.

Young, M.D., Wakefield, M.J., Smyth, G.K., and Oshlack, A. (2010). Gene ontology analysis for RNA-seq: accounting for selection bias. *Genome Biol* 11**,** R14.
