# Supplementary material for: Zebrafish Chromosome 14 Gene Differential Expression in the fmr1hu2787 Model of Fragile X Syndrome
Source: Front Genet. 2021 May 31;12:625466. doi: 10.3389/fgene.2021.625466 (PMC8203322; doi:10.3389/fgene.2021.625466)
Supplement: Supplementary file 3 [file Data_Sheet_3.pdf]

Supplementary Data File 3: Quantitative PCR (qPCR) analysis of relative chromosome abundance

Table S9. PCR primers for qPCR on genomic DNA

Target gene	Chromosome	Primer sequence
rpl13	7	5'-TGGAATGGATGAATAGGTTTTTA-3'
		5'-CTCTCTTCTGCCAGTCTTTATGA-3'
psen1	17	5'-CTACACACAGAAGGACGGACAGC-3'
		5'-CCATCCCTAAACTGCTCCTACT-3'
fmr1	14	5'-TCCAGGACCAGGAGGCTGTA-3'
		5'-CCCACTCTTGCCAATGACTTTTC-3'

Results

Table S10. The ratio of relative quantities between chromosomes

		**Genotype, Batch number** (Note: + denotes wild type *fmr1* allele, *fmr1*$^{-}$ denotes *fmr1*^{hu2787})					
		+/+, 1	+/+, 2	+/+, 3	*fmr1*$^{-}$/*fmr1*$^{-}$, 1	*fmr1*$^{-}$/*fmr1*$^{-}$, 2	*fmr1*$^{-}$/*fmr1*$^{-}$, 3
**Ratio**	*psen1/rpl13*	0.97625561	0.985557459	0.98787442	0.844421676	1.01865661	0.919412757
	fmr1/rpl13	1.046886584	0.96111932	0.971418831	0.907239527	1.026082501	1.005627511

Here, the quantities of chromosome 14 (*fmr1*) were normalized by the quantities of chromosome 7 (*rpl13*) (Table S10). The relative quantities, *fmr1*/*psen1* ratios, are close to 1 in all the samples and do not show any differences between wild-type samples and *fmr1* samples. If there is an extra copy of chromosome 14, it is expected to achieve a *fmr1*/*psen1* ratio of around 1.5. In conclusion, the quantity of chromosome 14 is the same as for the other chromosomes, so there is no evidence for the existence of additional copies of chromosome 14.

See Supplementary Data File 1 for raw qPCR data.
