# Supplementary material for: Zebrafish Chromosome 14 Gene Differential Expression in the fmr1hu2787 Model of Fragile X Syndrome
Source: Front Genet. 2021 May 31;12:625466. doi: 10.3389/fgene.2021.625466 (PMC8203322; doi:10.3389/fgene.2021.625466)
Supplement: Supplementary file 4 [file Data_Sheet_4.pdf]

Supplementary Data File 4: Analysis of allelic diversity in differentially expressed genes

Following identification of variants using the GATK workflow for RNAseq short variant discovery (See Materials & Methods section), variants were further processed to explore differences in allele proportions. Variant Call Format (VCF) files were merged and converted into a Genomic Data Structure (GDS) object using the SeqArray package. For each genomic position where a non-reference allele was detected (GRCz11, Ensembl version 94), allele counts were extracted and summed based on genotype. Genomic positions were restricted to only exonic regions. Particular genes of interest were classified into three separate groups to observe potential patterns of eQTL effects that may explain the enrichment of DE genes on Chromosome 14. Differences in allelic proportions were compared between genotypes within each group, and overall patterns were also compared between groups. The first group of genes consisted of the 12 DE genes on Chromosome 14. These were compared to a negative control set of 12 non-DE genes also on Chromosome 14. Each negative control gene was chosen by taking a DE gene from the first group, looking at the 25 genes either side by genomic position, and randomly sampling one gene that satisfies the criteria of a Benjamini-Hochberg adjusted p-value > 0.80 and an average expression > 2 $\log_2$ counts per million. The final set of genes analysed was the remaining 9 DE genes that do not lie on Chromosome 14. All three sets of genes were explored visually by plotting allelic proportions at each detected variant position (See Figures S9 and S10). The code used to perform this analysis is available at https://github.com/baerlachlan/210305_fmr1_snv.

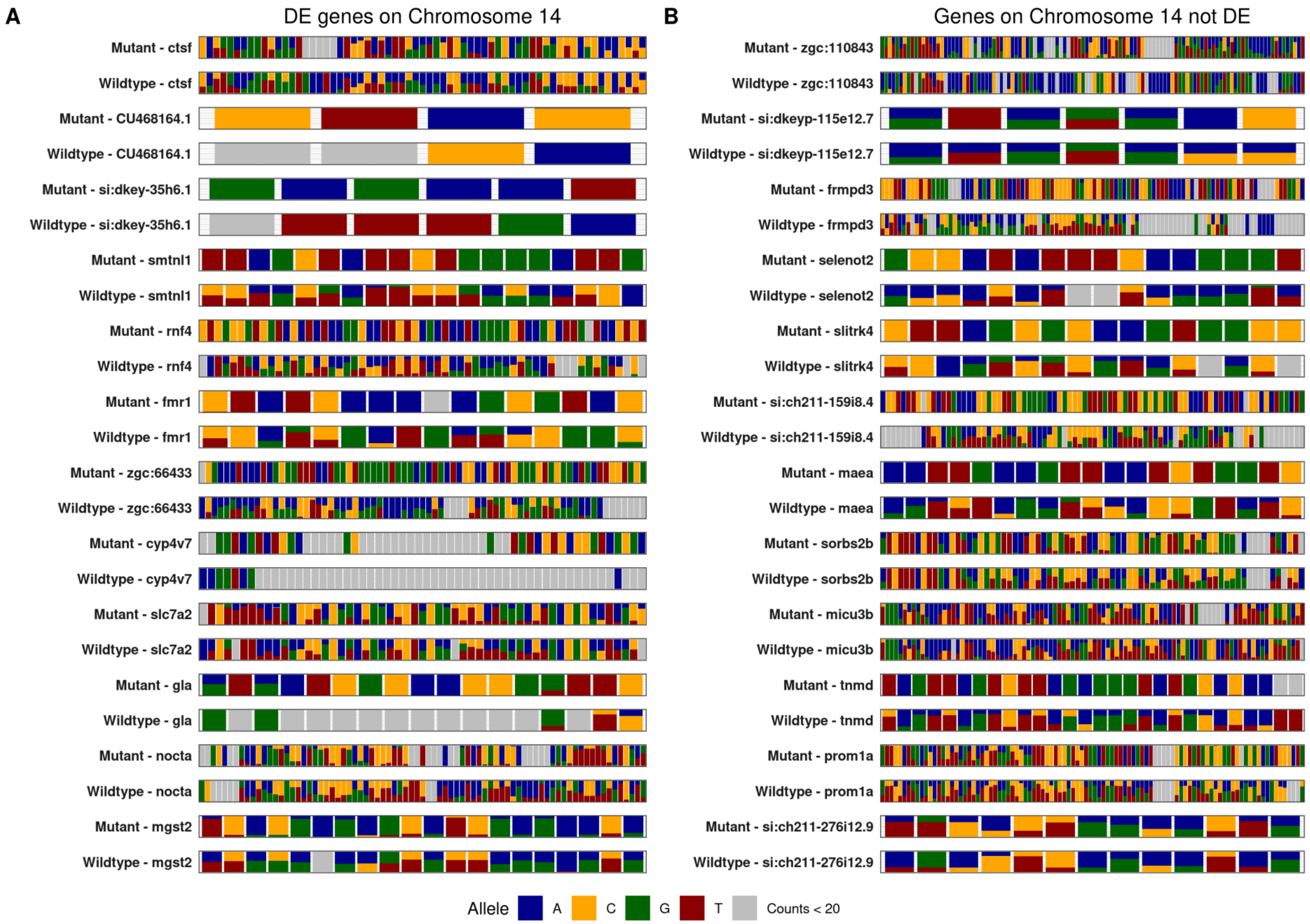
A
DE genes on Chromosome 14
B
Genes on Chromosome 14 not DE
Mutant - ctsf
Wildtype - ctsf
Mutant - CU468164.1
Wildtype - CU468164.1
Mutant - si:dkey-35h6.1
Wildtype - si:dkey-35h6.1
Mutant - smtnl1
Wildtype - smtnl1
Mutant - rnf4
Wildtype - rnf4
Mutant - fmr1
Wildtype - fmr1
Mutant - zgc:66433
Wildtype - zgc:66433
Mutant - cyp4v7
Wildtype - cyp4v7
Mutant - slc7a2
Wildtype - slc7a2
Mutant - gla
Wildtype - gla
Mutant - nocta
Wildtype - nocta
Mutant - mgst2
Wildtype - mgst2
Mutant - zgc:110843
Wildtype - zgc:110843
Mutant - si:dkeyp-115e12.7
Wildtype - si:dkeyp-115e12.7
Mutant - frmpd3
Wildtype - frmpd3
Mutant - selenot2
Wildtype - selenot2
Mutant - slitrk4
Wildtype - slitrk4
Mutant - si:ch211-159i8.4
Wildtype - si:ch211-159i8.4
Mutant - maea
Wildtype - maea
Mutant - sorbs2b
Wildtype - sorbs2b
Mutant - micu3b
Wildtype - micu3b
Mutant - tnmd
Wildtype - tnmd
Mutant - prom1a
Wildtype - prom1a
Mutant - si:ch211-276i12.9
Wildtype - si:ch211-276i12.9
Allele
A
C
G
T
Counts < 20

Figure S9: Gene allele proportions of detected variants by genotype for A) DE genes on Chromosome 14 and B) Non-DE genes on Chromosome 14. For each panel, genes are ordered vertically based on chromosomal position relative to *fmr1*. Each bar represents a singular base position where a variant was detected. Genotypes are plotted directly above/beneath each other to allow for direct comparison at each variant position. Similarly, DE and non-DE genes that have similar genomic positions are plotted side-by-side horizontally to allow for comparison between panels. The proportion of A nucleotides are represented in blue, C nucleotides are yellow, G nucleotides are green and T nucleotides are red. Positions with less than 20 total counts are represented in grey as their proportions cannot be estimated confidently.

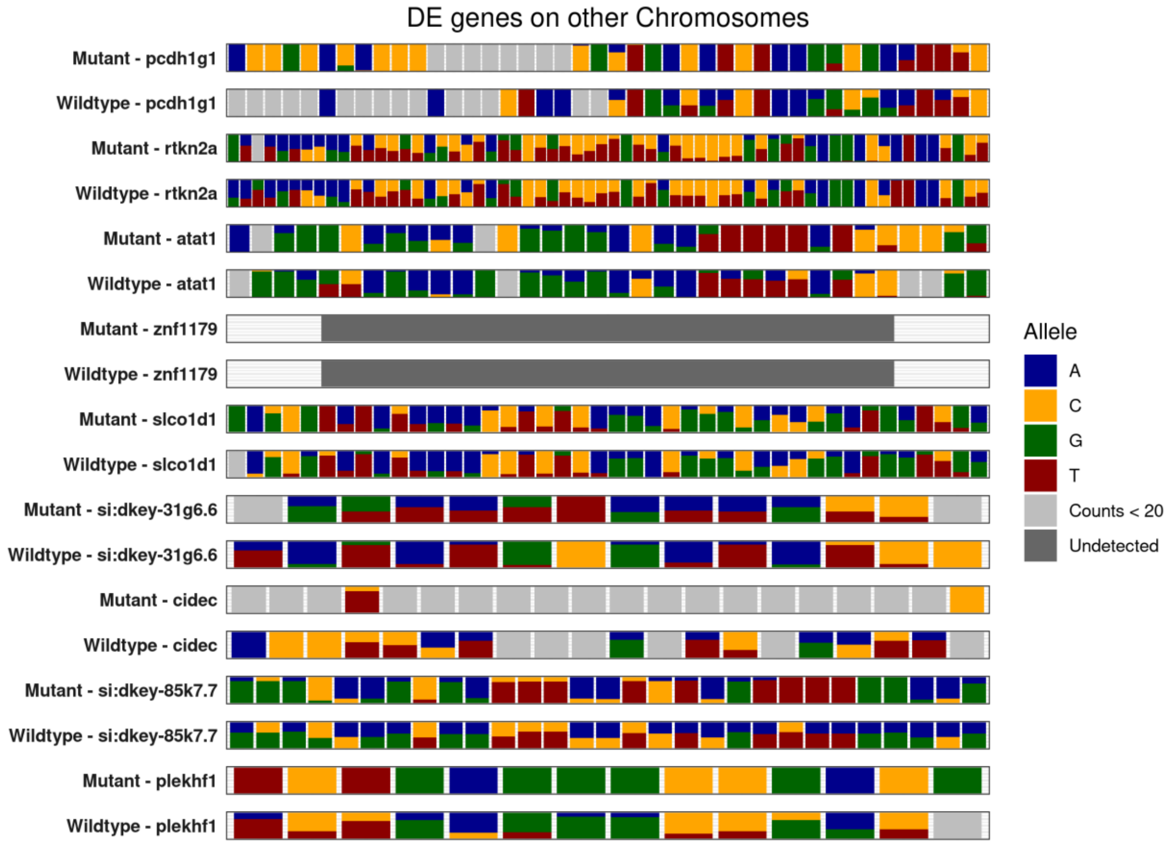

Figure S10: Gene allele proportions of detected variants by genotype for DE genes not on Chromosome 14. Each bar represents a singular base position where a variant was detected. Genotypes are plotted directly above/beneath each other to allow for direct comparison at each variant position. The proportion of A nucleotides is represented in blue, while C nucleotides are yellow, G nucleotides are green and T nucleotides are red. Positions with less than 20 total counts are represented in light grey as their proportions cannot be estimated with confidence. Genes where no non-reference alleles were detected are represented in dark grey.
