# Supplementary material for: Zebrafish Chromosome 14 Gene Differential Expression in the fmr1hu2787 Model of Fragile X Syndrome
Source: Front Genet. 2021 May 31;12:625466. doi: 10.3389/fgene.2021.625466 (PMC8203322; doi:10.3389/fgene.2021.625466)
Supplement: Supplementary file 5 [file Data_Sheet_5.pdf]

Supplementary Data File 5: Zebrafish transcripts with sequence homology to *fmr1*

The Ensembl BLAST server (http://asia.ensembl.org/Danio_rerio/Tools/Blast) was used to search all sequences across the zebrafish genome and transcriptome (Ensembl version 101), with parameters set for distant homology to the cDNA sequence of *fmr1* (ENSDART00000123434.3). Only results with an E-value < 1 were retained.

The BLAST results for *fmr1* are summarised in **Table S11**.

Table S11: Summary of BLAST matches for *fmr1*

BLAST Hit	Description	Total Matches	Longest Match
fxr1	fragile X mental retardation, autosomal homolog 1	10	1359
fxr2	fragile X mental retardation, autosomal homolog 2	16	977
nr2f6a	nuclear receptor subfamily 2, group F, member 6a	1	75
ZBTB26	zinc finger and BTB domain containing 26	1	72
nat10	N-acetyltransferase 10	1	46
rybpa	RING1 and YY1 binding protein a	1	43
ctdnep1b	CTD nuclear envelope phosphatase 1b	1	38
gnb1b	guanine nucleotide binding protein (G protein), beta polypeptide 1b	1	35
acvr2aa	activin A receptor type 2Aa	1	34
ube2d2	ubiquitin-conjugating enzyme E2D 2 (UBC4/5 homolog, yeast)	1	31

An exploration of the relationship between sequence similarity and upregulation, based on several similarity thresholds, revealed no significant correlation (**Figure S9**).

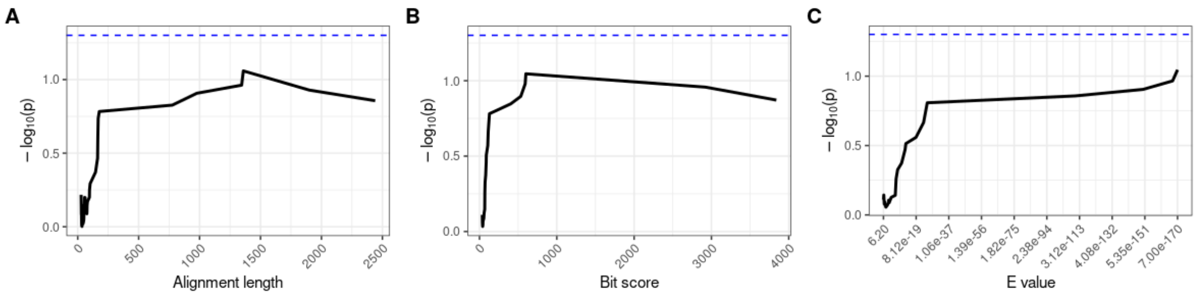

Figure S11: Impact of various thresholds of three different alignment-quality parameters of BLASTn on the significance of the observed correlation between upregulation and sequence similarity. The y-axis of each diagram shows the negative $\log_{10}$ *p*-value and the *x*-axis shows the respective alignment-quality parameter. A *p*-value of 0.05 is represented as a horizontal dashed blue line in each diagram. *p*-values were calculated by bootstrapping random subsamples (see El-Brolosy et al. (El-Brolosy et al., 2019) methods section 'Sequence similarity and subsampling analyses') and

were not adjusted for multiple testing. **A)** Alignment length describes the length of sequence satisfying similarity criteria of BLASTn. **B)** Bit score is a value that represents how good an alignment is based on alignment quality and length. **C)** E value describes the probability of a match resulting from chance.

Although not the subject of investigation, two of the genes detected as differentially expressed (*smtnl1* and *CU468164.1*) were analysed using BLAST as a reverse viewpoint on the experiment. Neither of the genes contained matches to *fmr1* (**Tables S12, S13**).

**Table S12: Summary of BLAST matches for *smtnl1***			
**BLAST Hit**	**Description**	**Total Matches**	**Longest Match**
smtna	smoothelin a	1	297
smtnb	smoothelin b	4	258
si:ch211-195o20.7	si:ch211-195o20.7	1	173
CU929178.1	NUL	1	134
nefla	neurofilament, light polypeptide a	3	132
psd2	pleckstrin and Sec7 domain containing 2	1	92
si:dkey-20i20.10	si:dkey-20i20.10	1	88
taf3	TAF3 RNA polymerase II, TATA box binding protein (TBP)-associated facto	2	84
tgfbr2a	transforming growth factor beta receptor 2a	1	78
ppp1r37	protein phosphatase 1, regulatory subunit 37	1	76
brd3b	bromodomain containing 3b	3	63
cmya5	cardiomyopathy associated 5	2	61
chd5	chromodomain helicase DNA binding protein 5	1	60
chd2	chromodomain helicase DNA binding protein 2	2	58
zgc:175284	zgc:175284	2	54
zgc:173726	zgc:173726	6	52
map6a	microtubule-associated protein 6a	2	52
si:dkey-20i20.9	si:dkey-20i20.9	1	52
znf1170	zinc finger protein 1170	1	52
stab2	stabilin 2	4	51
znf1166	zinc finger protein 1166	3	51
igfn1.1	immunoglobulin-like and fibronectin type III domain containing 1, tandem duplicate 1	1	49
zswim8	zinc finger, SWIM-type containing 8	4	47
kcnma1a	potassium large conductance calcium-activated channel, subfamily M, alpha member 1a	3	44
gpbp1l1	GC-rich promoter binding protein 1-like 1	2	44
foxn2b	forkhead box N2b	1	43

polr2f	polymerase (RNA) II (DNA directed) polypeptide F	1	42
lmod1a	leiomodin 1a (smooth muscle)	1	41
nktr	natural killer cell triggering receptor	1	41
im:7152348	im:7152348	1	39
jak2b	Janus kinase 2b	1	35
nop58	NOP58 ribonucleoprotein homolog (yeast)	1	35
zfr2	zinc finger RNA binding protein 2	2	29
chd3	chromodomain helicase DNA binding protein 3	3	23

Table S13: Summary of BLAST matches for *CU468164.1*

BLAST Hit	Description	Total Matches	Longest Match
si:ch211-262h13.3	si:ch211-262h13.3	5	268
si:ch211-244k5.1	si:ch211-244k5.1	1	267
si:dkey-242h9.3	si:dkey-242h9.3	1	70
or117-1	odorant receptor, family F, subfamily 117, member 1	1	53
cib1	calcium and integrin binding 1 (calmyrin)	2	39
im:7147486	im:7147486	2	35
ppp1r18	protein phosphatase 1, regulatory subunit 18	1	35

Conclusion:

No clear evidence was found for transcriptional adaptation in this dataset.
Alternative strategies were investigated briefly using pair-wise alignments between two other genes identified to be differentially expressed, but no compelling evidence was found.

Reference:

El-Brolosy, M.A., Kontarakis, Z., Rossi, A., Kuenne, C., Gunther, S., Fukuda, N., Kikhi, K., Boezio, G.L.M., Takacs, C.M., Lai, S.L., Fukuda, R., Gerri, C., Giraldez, A.J., and Stainier, D.Y.R. (2019). Genetic compensation triggered by mutant mRNA degradation. *Nature* 568**,** 193-197.
